# Prenatal Exposure to Chemical Mixtures and Cognitive Flexibility among Adolescents

**DOI:** 10.3390/toxics9120329

**Published:** 2021-12-02

**Authors:** Anna V. Oppenheimer, David C. Bellinger, Brent A. Coull, Marc G. Weisskopf, Susan A. Korrick

**Affiliations:** 1 Department of Environmental Health, Harvard T.H. Chan School of Public Health, Boston, MA 02115, USA; david.bellinger@childrens.harvard.edu (D.C.B.); bcoull@hsph.harvard.edu (B.A.C.); mweissko@hsph.harvard.edu (M.G.W.); susan.korrick@channing.harvard.edu (S.A.K.); 2 Department of Psychiatry, Boston Children’s Hospital, Boston, MA 02115, USA; 3 Department of Biostatistics, Harvard T.H. Chan School of Public Health, Boston, MA 02115, USA; 4 Department of Epidemiology, Harvard T.H. Chan School of Public Health, Boston, MA 02115, USA; 5 Channing Division of Network Medicine, Brigham and Women’s Hospital, Harvard Medical School, Boston, MA 02115, USA

**Keywords:** prenatal exposures, chemical mixtures, organochlorines, metals, cognitive flexibility, executive function, adolescent neurodevelopment

## Abstract

Cognitive flexibility, the ability to smoothly adapt to changing circumstances, is a skill that is vital to higher-level executive functions such as problem-solving, planning, and reasoning. As it undergoes substantial development during adolescence, decrements in cognitive flexibility may not become apparent until this time. There is evidence that prenatal exposure to individual chemicals may adversely impact executive functions in children, but few studies have explored the association of co-exposure to multiple chemicals with cognitive flexibility specifically among adolescents. We investigated this association among a diverse group of adolescents living near a Superfund site in New Bedford, Massachusetts. Specifically, using Bayesian kernel machine regression (BKMR) and multivariable regression analyses, we investigated the association of biomarkers of prenatal exposure to organochlorines (DDE, HCB, PCBs) and metals (lead, manganese) with cognitive flexibility, measured with four subtests of the Delis-Kaplan Executive Function System. In BKMR models, we observed adverse joint associations of the chemical mixture with two of the four cognitive flexibility subtests. In covariate-adjusted linear regression models, a two-fold increase in cord blood Mn was associated with poorer performance on two of the subtests: Trail-Making (scaled score difference = −0.60; 95% CI: −1.16, −0.05 points) and Color-Word Interference (scaled score difference = −0.53; 95% CI: −1.08, 0.01 points). These adverse Mn-cognitive flexibility associations were supported by the results of the BKMR. There was little evidence of effect modification by sex and some evidence of effect modification by a measure of social disadvantage, particularly for the associations between HCB and cognitive flexibility. This study is among the first to provide evidence of an adverse association of prenatal exposure to a chemical mixture with cognitive flexibility in adolescence.

## 1. Introduction

Cognitive flexibility is one of the core executive functions along with inhibition and working memory that serve as building blocks of higher-level cognitive processes such as problem-solving [1]. It involves the ability to shift perspectives, adapt to changing circumstances, and switch flexibly between tasks [1]. Development of the core executive function skills such as inhibitory control and working memory begins during early childhood; however, because cognitive flexibility builds on these skills, it evolves later with substantial development occurring during adolescence [1,2]. Cognitive flexibility facilitates a number of important skills including effective problem solving, emotional regulation, and resilience [3]. Better performance on cognitive flexibility tasks has been found to predict academic achievement, particularly reading, mathematical, and writing skills in a study of college students [4]. Meanwhile, impairments in cognitive flexibility skills play a role in multiple behavioral and psychiatric disorders including attention-deficit hyperactivity disorder, anxiety, depression, bipolar disorder, obsessive-compulsive disorder, and schizophrenia [5,6,7,8,9].

In utero, the developing brain undergoes a period of rapid neurological growth, yet it is not well-protected from environmental toxicants during this time and is therefore highly sensitive to injury from such exposures [10,11,12]. There is some evidence of associations of prenatal exposures to polychlorinated biphenyls (PCBs) and lead (Pb) with poor performance on tasks assessing cognitive flexibility in mid-childhood [13,14]. Specifically, Pb levels measured in maternal erythrocytes during pregnancy were adversely associated with cognitive flexibility among 7-year-olds as measured by a parent-rated behavioral checklist [13]. In addition, cord serum PCB concentrations were associated with decrements in the Wisconsin Card Sorting Test (WCST), a psychometric test of cognitive flexibility, among 11-year-olds in a high fish-eating population [14].

Prenatal exposure to chemical contaminants has also been studied in relation to other components of executive function and general executive function in childhood and adolescence. Prenatal exposure to dichlorodiphenyldichloroethylene (DDE), hexachlorobenzene (HCB), and PCBs, individually, have each been adversely associated with both psychometric testing and behavioral checklist assessments of inhibition and working memory in early to mid-childhood, though these findings have not been consistent across studies [14,15,16,17,18,19,20,21]. Prenatal exposure to metals such as Pb and manganese (Mn) has also been implicated as harmful to inhibition and working memory in age groups ranging from early childhood through adolescence [13,22,23]. There are few studies of the association of prenatal methylmercury (MeHg) or arsenic (As) exposure with executive function. However, cross-sectional studies have found adverse correlations between As exposure and working memory, switching attention, and problem-solving among children ranging from ages 3 through 16 years [24,25,26,27]. 

There is also evidence that exposure to chemical contaminants rarely occurs independently, and multiple chemicals may interact to induce differing health effects than individual exposures [28]. Despite this, there are no studies investigating the association of a chemical mixture with cognitive flexibility specifically. Of the few studies that have assessed the relation of multiple chemical exposures with other executive functions, some have found evidence of interactions between chemicals [29,30], though others have not [31]. In one prospective cohort study, there was a stronger adverse association between current blood Pb and an inhibition task among school-aged children with lower current MeHg and PCB blood concentrations [29]. Similarly, in another prospective cohort study, there was an adverse association between cord blood Pb and a working memory task among those with low cord blood MeHg [30]. Meanwhile, in a cross-sectional study demonstrating an association of blood Mn and As with working memory among school-age children, researchers did not find evidence of an interaction between the two chemicals in this association [31].

In summary, few studies have assessed the impact of co-exposure to these chemicals and none specifically on cognitive flexibility. Given that substantial development of cognitive flexibility occurs in adolescence and therefore alterations in this skill may not become apparent until this time, the goal of this study was to assess the relation of prenatal exposure to a chemical mixture composed of organochlorines and metals with cognitive flexibility among adolescents. In addition, given that previous studies of the association between prenatal exposures to individual neurotoxicants and executive function have found evidence of effect modification by sex and socioeconomic stressors [18,19,23,32,33,34], we assessed effect modification by sex and measures of social disadvantage in this study.

## 2. Materials and Methods

### 2.1. Study Population

The New Bedford Cohort (NBC) study is a longitudinal birth cohort study designed to assess the impacts of prenatal chemical pollutant exposures on child health and development among families living near the New Bedford Harbor. The New Bedford Harbor was designated a Superfund site in 1982 due to PCB contamination from local industry [35]. Between 1993 and 1998, 788 mother-infant pairs were recruited into the NBC study shortly after the infant’s birth. Recruited mothers were at least 18 years old, spoke English or Portuguese, and were living in one of the four towns surrounding the New Bedford Harbor throughout their pregnancy. Infants born by cesarean section or who were too ill to undergo study neonatal examinations were excluded from the study. Study participants have been followed periodically since birth. At an adolescent follow-up that occurred between 2008 and 2014, 528 children (median age 15.5, range 13.9–17.9 years) completed neurodevelopmental assessments including measures of cognitive flexibility. A subset of 373 of the NBC adolescents had complete data on cognitive flexibility outcomes, covariates, and biomarkers of prenatal exposure to DDE, HCB, PCBs, Pb, and Mn. 235 participants had additional data on biomarkers of prenatal exposure to MeHg and As.

### 2.2. Chemical Exposure Assessment

DDE, HCB, and 51 PCB congeners were measured in cord serum, Pb and Mn were measured in cord blood, and MeHg and As were measured in maternal hair and toenails, respectively. Cord blood was collected at birth, while maternal hair and toenails were collected, on average, two weeks postpartum. With this collection time, the proximal maternal hair sample was used to estimate exposures during the third trimester of pregnancy, while maternal toenails were used to estimate exposures throughout the entirety of the pregnancy. PCB exposure was estimated using the sum of the 4 most prevalent congeners (ΣPCB_4_: 118, 138, 153, 180) due to their minimal measurement error and common usage to assess congener-specific effects in other population-based studies. Details regarding the collection, storage, and exposure analysis methods of these biomarkers have been described elsewhere [36,37]. Briefly, cord serum was analyzed for DDE, HCB, and PCBs using gas chromatography with electron capture detection [36], while cord blood was analyzed for Pb using isotope dilution inductively coupled plasma mass spectrometry (ICP-MS, Sciex Elan 5000, Perkin Elmer, Norwalk, CT, USA) and for Mn using external calibration on a dynamic reaction cell-inductively coupled plasma-mass spectrometer (DRC-IPC-MS, Elan 6100, Perkin Elmer, Norwalk, CT, USA). Hair was analyzed for total Hg, a reasonable proxy for MeHg [38], using a DMA-80 Direct Mercury analyzer (Milestone, Inc., Monroe, CT, USA), and toenails were analyzed for As using an external calibration method on a dynamic reaction cell-inductively coupled plasma-mass spectrometer (Agilent 7700x ICP-MS, Santa Clara, CA, USA). For the organochlorines, within-batch coefficients of variation ranged from 5% to 7.5% and between-batch coefficients of variation ranged from 20% to 39% over the 5 years of analysis, reflecting high reproducibility for organics analyses [36]. For Pb and Mn, quality control (QC) monitoring, procedural blanks, duplicates, spiked samples, standard reference material (NIST SRM 955b Pb in blood; NIST SRM 1643d trace elements in water), and biological reference material (ICP03B-05 and ICP03B-02 multi-elements in human blood from INSPQ/Laboratoire de Toxicologie, Quebec, QC, Canada) were used. Recovery rates for QC and spiked samples were 90–110% and precision >95%. Finally, for MeHg and As, QC procedures included daily calibration verification, procedural blanks, and certified reference material (GBW 09101 human hair, Shanghai Institute of Nuclear Research, Academia Sinica, Shanghai, China). Recovery rates for QC standards were 90–110% and precision >95% for hair MeHg, while the coefficients of variation for reference standards were <15% for toenail As [37,39]. The detection limits for DDE, HCB, and PCBs ranged from 0.001 ng/g to 0.07 ng/g serum, for Pb and Mn was 0.02 µg/dL, for MeHg was 50 ng/g of hair, and for As was 0.03 ng/g of the toenail.

### 2.3. Cognitive Flexibility Assessment

Cognitive flexibility was measured using four subtests of the Delis-Kaplan Executive Function System (D-KEFS) [40], which were administered by a trained study examiner at the NBC study adolescent (~15-year) follow-up visit. The four subtests included: Trail-Making: Number-Letter Switching condition, Verbal Fluency: Category Switching condition, Design Fluency: Filled Dots and Empty Dots Switching condition, and Color-Word Interference: Inhibition/Switching condition. In Trail-Making: Number-Letter Switching, participants connected numbers then letters switching in the correct order (e.g., from A to 1 to B to 2). Performance was measured by the completion time scaled score, total errors raw score (the sum of set loss, sequencing, and time-discontinue errors), and an overall performance measure that combined both completion time and total errors raw scores to account for both speed and accuracy of test performance. This derived measure created a potentially more comprehensive representation of overall performance than is possible by assessing measures of speed and accuracy separately, as is usually done. Specifically, the overall performance measure was defined as a dichotomous measure where the best performance group included those who performed better than the population median score for both dimensions (total completion time raw score <67 s and total errors raw score <1 error) and the poor performance group included the remaining participants. In Verbal Fluency: Category Switching, participants alternated between saying words from two different categories as quickly as possible for 60 s. Performance was measured with the switching accuracy scaled score and the total errors raw score, which was defined as the sum of the total set loss errors and the total repetition errors. In Design Fluency: Filled Dots and Empty Dots Switching, participants drew designs by switching connections between filled and empty dots. Performance was measured using the total correct scaled score and the total errors raw score, which was defined as the sum of the total set loss designs and the total repeated designs raw scores. In Color-Word Interference: Inhibition/Switching, participants switched between naming font colors and reading words that named a color but were printed in a different colored font. Similar to Trail-Making, performance was measured using the completion time scaled score and total errors raw score (the sum of uncorrected and self-corrected errors) as well as a derived dichotomized performance measure to account for both speed and accuracy of test performance where the best performance group included those who performed better than the population median for both dimensions (total completion time raw score <59 s and total errors raw score <2 errors) and the poor performance group included the remaining participants. For all measures, scaled scores were age-standardized to a mean of 10 and a standard deviation of 3. Of note, higher scaled scores mean better performance. Raw scores were used for total errors because scaled scores were unavailable and for defining dichotomized performance measures (for Trail Making and Color Word Interference) so that the speed and accuracy measures were on the same scale. Higher error and completion time raw scores mean worse performance.

### 2.4. Covariate Assessment

Participant demographic, health, and lifestyle information were obtained via medical record review and parental and child self-reported questionnaires at multiple time points. Hospital medical records were reviewed to obtain information regarding the infant’s race/ethnicity, birthweight, gestational age, and newborn exam as well as the mother’s pregnancy and delivery course. At a home visit that took place two weeks postpartum, participating mothers completed a questionnaire regarding maternal socio-demographics, medical history, pregnancy lifestyle (diet, smoking, and alcohol use), drug use, and infant feeding. At a 15-year follow-up assessment, in addition to updating medical and demographic information, a home visit took place to assess the quality of the child’s home environment and parent-child relationship using the Home Observation for Measurement of the Environment (HOME) assessment instrument [41] and maternal IQ was measured using the Kaufman Brief Intelligence Test (KBIT) [42].

In this study, we defined social disadvantage using a prenatal social disadvantage index (PNSDI) composed of the sum of five adverse social/economic exposures at the time of the child’s birth where the presence of each of the following risk factors was assigned a value of 1 and absence a value of 0: mother unmarried, mother’s education as a high school graduate or less, father’s education as a high school graduate or less, annual household income less than $20,000, and mother’s age at birth less than 20 years.

### 2.5. Statistical Analysis

We used directed acyclic graphs (DAGs) to select potential covariates. The DAG was informed by a literature review of potential confounders of the relationships of prenatal organochlorine and metal exposures with neurodevelopment, as well as covariates that had previously predicted cognition outcomes in the NBC. The following covariates were included in the final models: adolescent race/ethnicity, sex, age at exam, and HOME score; maternal marital status at birth, IQ, seafood consumption and smoking during pregnancy; maternal and paternal education and household income at child’s birth; and examiner. Characteristics of participants who were included in analyses were compared to those not included using t-tests, Wilcoxon Rank Sum tests, and chi-square tests where appropriate.

To analyze the association between a five-chemical mixture composed of DDE, HCB, ΣPCB_4_, Pb, and Mn and multiple measures of cognitive flexibility, we first log_2_-transformed exposures to reduce the influence of extreme values. Next, we used Bayesian kernel machine regression (BKMR) as an exploratory technique to examine potential non-linear relationships and interactions between chemical exposures and continuous measures of cognitive flexibility using the scaled scores. BKMR, an exposure-response surface estimation technique, can model the relationship between multiple exposures and an outcome using a flexible exposure-response function, *h* [43]. In this analysis, *h* was estimated using a Gaussian Kernel, which can capture many underlying functional forms [43]. We did not analyze the error scores for D-KEFS cognitive flexibility measures using BKMR as they have distributions consistent with count data which cannot currently be accommodated as an outcome in BKMR analyses [43].

Using BKMR, we were able to identify non-linear exposure-outcome associations and interactions between exposures, which were then used to inform the specification of standard parametric linear regression models. Specifically, to assess non-linearities, we visually examined plots of the estimated exposure-response functions and 95% credible intervals of DDE, HCB, ΣPCB_4_, Pb, and Mn with cognitive flexibility scaled scores while the remaining exposures were assigned their median value. If the exposure-response function appeared non-linear, we included a quadratic term for the exposure in the model and used a likelihood ratio test with two degrees of freedom to assess the significance of the exposure overall. To assess interactions, we visually examined plots of the estimated exposure-response functions between one of the five main exposures (DDE, HCB, ΣPCB_4_, Pb, Mn) and cognitive flexibility scaled scores, where a second exposure was fixed at varying levels and all of the remaining exposures were assigned to their median value. We assumed no interaction in cases where the slope of each chemical was similar at varying levels of the other chemicals. In most cases, BKMR supported the use of linear regression models with no interactions between exposures. We also used BKMR to assess the joint association between the chemical mixture and each cognitive flexibility scaled score by measuring the association between the outcomes and chemical mixture levels at various percentiles compared to their median levels. BKMR analyses were conducted using the *bkmr* package in R [44].

As Trail-Making completion time, Verbal Fluency switching accuracy, Design Fluency total correct, and Color-Word completion time scaled scores were normally distributed, we used ordinary least squares (OLS) to fit multivariable linear regression models to estimate their association with the five-chemical mixture. All five exposures (DDE, HCB, ΣPCB_4_, Pb, and Mn) were simultaneously included in the linear regression models along with the previously mentioned covariates. We then assessed effect modification of exposure-outcome associations by sex and by prenatal social disadvantage by first including chemical-sex or chemical-PNSDI interaction terms in the models and assessing the statistical significance of the interaction terms. We then analyzed sex-specific and PNSDI-specific associations using sex- and PNSDI-stratified linear regression models. In PNSDI-stratified models, we compared chemical associations among participants who had a PNSDI of 3 or more (more prenatal social disadvantage) to those who had a PNSDI of less than 3 (less prenatal social disadvantage). A cut-off of 3 was selected as it was more predictive of other indicators of social disadvantage such as the HOME score than other cut-offs while maintaining enough power to conduct stratified analyses.

As a secondary analysis, we analyzed the association between the five-chemical mixture and cognitive flexibility error scores, specifically Trail-Making, Verbal Fluency, Design Fluency, and Color-Word total errors raw scores. Once again, all five exposures of interest were included in the models together with covariates. All error scores had distributions consistent with count data, but Trail-Making, Verbal Fluency, and Design Fluency total error raw scores were consistent with over-dispersed count data. As negative binomial regression is a more flexible approach than Poisson regression and is appropriate for both count data and over-dispersed count data, it was used to assess the relationship of all error scores with the chemical mixtures. Next, logistic regression was used to assess the relationship of the chemical mixture with the derived dichotomous measures of overall Trail-Making and Color-Word interference performance. We assessed the odds of being in the poor compared to the best performance (reference) group.

Finally, we used inverse probability weighting (IPW) for censoring to account for potential selection bias due to loss to follow-up [45]. In IPW, individuals in the analysis group are weighted based on the inverse of the probability of their being included in the analysis given their particular exposure and covariate values, creating a pseudo-population that represents the original source population that was recruited to the NBC at birth. Biomarker levels of DDE, HCB, ΣPCB_4_ and Pb and socio-demographic characteristics of the mother at birth such as education and household income and child characteristics such as race/ethnicity and sex were used in the IPW missingness model based on how well they predicted loss to follow-up in this analysis and other studies reported in the literature. In this analysis, we used stabilized weights trimmed at the 2.5th and 97.5th percentile to improve efficiency and minimize the influence of extreme weights [45].

As another secondary analysis, we added biomarkers of MeHg and As to our chemical mixture and analyzed the association of DDE, HCB, ΣPCB_4_, Pb, Mn, MeHg, and As with cognitive flexibility. Many participants had missing information for these two exposure biomarkers as maternal hair and toenail samples were collected at a home visit two weeks postpartum, rather than at birth like the remaining exposures. To assess the relationship between the seven-chemical mixture and cognitive flexibility, we once again used BKMR to assess non-linear associations, interactions, and joint associations and to inform subsequent parametric models; multivariable linear regression to assess cognitive flexibility scaled scores and IPW for censoring to account for potential selection bias due to loss to follow-up. All statistics were conducted using R version 3.6.0 [46].

## 3. Results

### 3.1. Study Population

Table 1 describes the outcome, exposure, and covariate measures of all NBC participants included in our main analysis [those who had complete 15-year data on cognitive flexibility outcomes, biomarkers of prenatal exposure to DDE, HCB, ΣPCB_4_, Pb, and Mn, and covariates (*n* = 373)], and those who were excluded from the main analysis. Participants in the main analysis group were generally socio-demographically diverse, however, those who were included in the analysis had characteristics consistent with greater sociodemographic and economic advantage compared to those with missing data. For example, included adolescents had parents who were more likely to have more than a high school education and an annual household income at or above $20,000 at the time of their birth than those who were excluded from the analyses. Included participants also had higher cord serum DDE and lower cord blood Pb levels than those who were excluded due to loss to follow-up or missing data. They also performed better on some tests of cognitive flexibility than those who were excluded.

### 3.2. Chemical Exposure Measures

Despite its residential proximity to the NBH Superfund site, DDE, HCB, ΣPCB_4_, Pb, and Mn levels in NBC study participants were similar to the general populations of the U.S. and Canada [47,48,49]. Among the 373 participants in the main analysis group, DDE and ΣPCB_4_ exposures were strongly correlated (Spearman *r* = 0.7), while HCB exposures was moderately correlated with both DDE and ΣPCB_4_ (Spearman *r* = 0.4 for both correlations). Meanwhile, Pb and Mn exposures were not well correlated with the organochlorines or with each other (Spearman *r* = 0–0.2).

### 3.3. Executive Function Measures

D-KEFS Trail-Making, Verbal Fluency, Design Fluency, and Color-Word Interference scaled scores were weakly positively correlated with each other (Spearman *r* = 0.2–0.4). As expected, total error raw scores (higher score reflects worse performance) were negatively correlated with scaled scores (higher score reflects better performance) (e.g., Trail-Making completion time and total errors Spearman *r* = −0.5). NBC D-KEFS cognitive flexibility scaled scores (Table 1) were lower than the standardized sample [mean (SD) of cognitive flexibility scaled scores = 10 (3)].

### 3.4. BKMR Analysis of Cognitive Flexibility Scaled Scores and Prenatal Chemical Mixture Exposures

**Figure 1:**
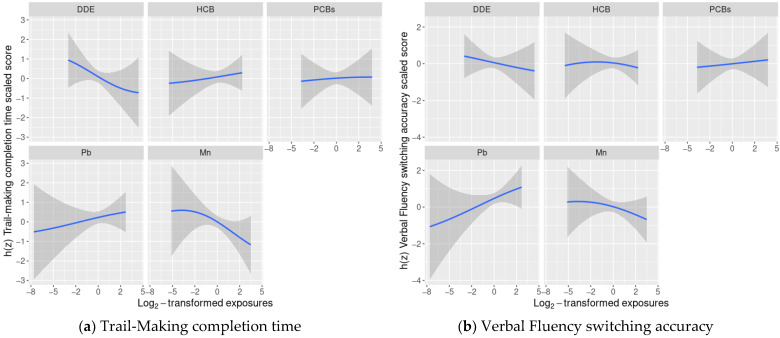

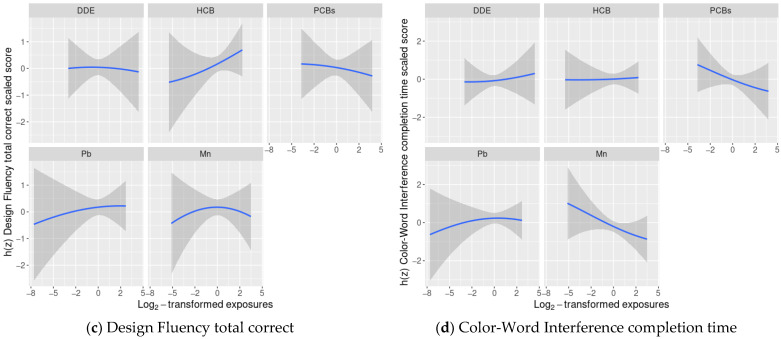
Estimated covariate-adjusted exposure-response functions and 95% credible intervals ^1^ between DDE, HCB, ΣPCB_4_, Pb, and Mn and Delis Kaplan Executive Function System (D-KEFS) cognitive flexibility scaled scores: (**a**) Trail-Making completion time; (**b**) Verbal Fluency switching accuracy; (**c**) Design Fluency total correct; and (**d**) Color-Word Interference completion time among adolescents in the main analysis group ^2^. In each plot, all of the remaining exposures are assigned their median value. (^1^ Exposures have been log2-transformed and models have been adjusted for child race, sex, age at exam, year of birth, and HOME score; maternal marital status at child’s birth, IQ, seafood consumption during pregnancy, and smoking during pregnancy; maternal and paternal education and annual household income at child’s birth, and study examiner. ^2^ Main analysis group: complete outcome, covariate, and prenatal exposure biomarker data for DDE, HCB, ΣPCB_4_, Pb, and Mn, *n* = 373. Abbreviations: DDE: dichlorodiphenyldichloroethylene; HCB: hexachlorobenzene; ΣPCB_4_: Sum of 4 PCB congeners (118, 138, 153, 180); Pb: lead; Mn: manganese).

Exploratory BKMR analyses of the association of a chemical mixture composed of DDE, HCB, ΣPCB_4_, Pb, and Mn with the cognitive flexibility outcomes indicated some potential non-linear relationships, such as Mn and Design Fluency (Figure 1), however, adding a quadratic term to the model was not supported by a likelihood ratio test with 2 degrees of freedom, therefore no quadratic terms were included in the final models.

**Figure 2:**
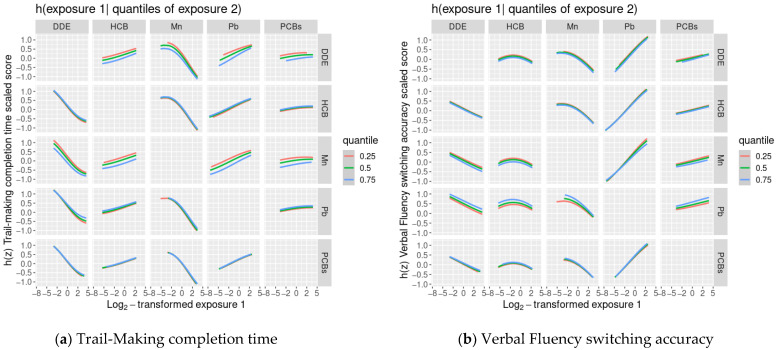

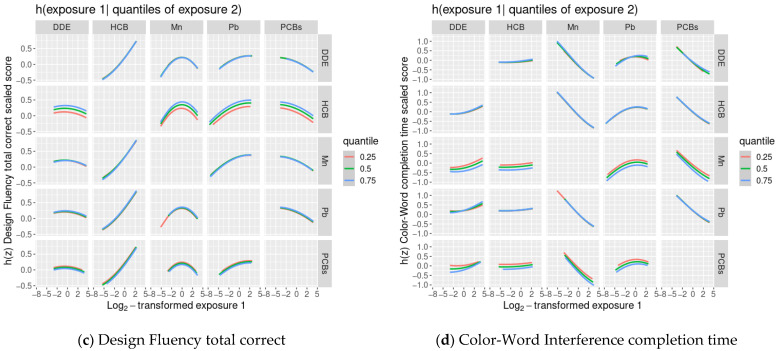
Covariate-adjusted exposure-response functions ^1^ between one of the 5 main exposures (DDE, HCB, ΣPCB_4_, Pb, Mn) and Delis-Kaplan Executive Function System (D-KEFS) cognitive flexibility scaled scores: (**a**) Trail-Making completion time; (**b**) Verbal Fluency switching accuracy; (**c**) Design Fluency total correct; and (**d**) Color-Word Interference completion time, where a second exposure is fixed at various quantiles., among adolescents in the main analysis group ^2^. In each plot, all of the remaining exposures are assigned to their median value. (^1^ Exposures have been log2-transformed and models have been adjusted for child race, sex, age at exam, year of birth, and HOME score; maternal marital status at child’s birth, IQ, seafood consumption during pregnancy, and smoking during pregnancy; maternal and paternal education and annual household income at child’s birth; and study examiner. ^2^ Main analysis group: complete outcome, covariate, and prenatal exposure biomarker data for DDE, HCB, ΣPCB_4_, Pb, and Mn, *n* = 373. Abbreviations: DDE: dichlorodiphenyldichloroethylene; HCB: hexachlorobenzene; ΣPCB_4_: Sum of 4 PCB congeners (118, 138, 153, 180); Pb: lead; Mn: manganese).

We did not find evidence of any other non-linear relationships or any interactions between chemicals (Figure 1 and Figure 2).

**Figure 3:**
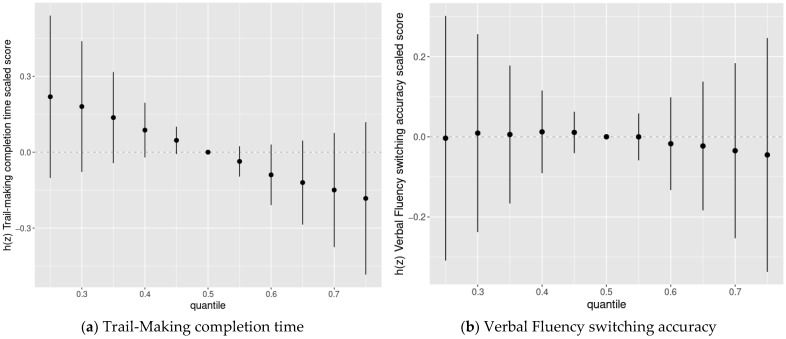

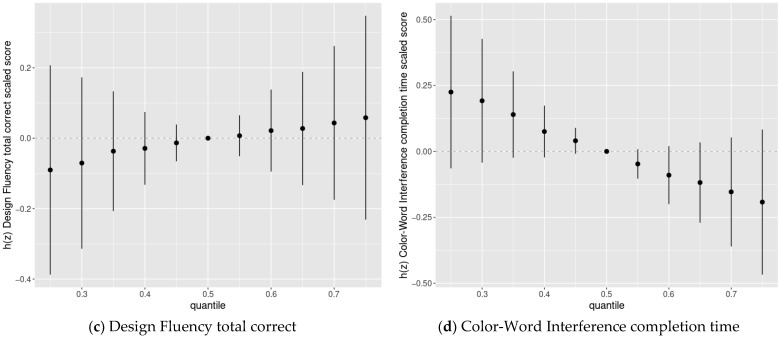
Joint association between the chemical mixture composed of DDE, HCB, PCBs, Pb, and Mn (estimates and 95% credible intervals ^1^) and the Delis-Kaplan Executive Function System (D-KEFS) cognitive flexibility scaled scores: (**a**) Trail-Making completion time; (**b**) Verbal Fluency switching accuracy; (**c**) Design Fluency total correct; and (**d**) Color-Word Interference completion time, comparing chemical mixture levels at various percentiles compared to a mixture with each component at its median level, among adolescents in the main analysis group ^2^. (^1^ Exposures have been log2-transformed and models have been adjusted for child race, sex, age at exam, year of birth, and HOME score; maternal marital status at child’s birth, IQ, seafood consumption during pregnancy, and smoking during pregnancy; maternal and paternal education and annual household income at child’s birth; and study examiner. ^2^ Main analysis group: complete outcome, covariate, and prenatal exposure biomarker data for DDE, HCB, ΣPCB_4,_ Pb, and Mn, *n* = 373. Abbreviations: DDE: dichlorodiphenyldichloroethylene; HCB: hexachlorobenzene; ΣPCB_4_: Sum of 4 PCB congeners (118, 138, 153, 180); Pb: lead; Mn: manganese).

We then used BKMR to assess the joint association of the chemicals with cognitive flexibility scaled scores. We found evidence of adverse joint associations of the five-chemical mixture with Trail-Making completion time scaled scores and Color-Word completion time scaled scores, but not Verbal Fluency switching accuracy scaled scores or Design Fluency total correct scaled scores (Figure 3).

### 3.5. Linear Regression Analyses of Cognitive Flexibility and Prenatal Chemical Mixture Exposures

In linear regression models adjusted for covariates and the remaining exposures, a twofold increase in cord blood Mn concentrations was associated with lower cognitive flexibility scaled scores on two subtests: Trail-Making (completion time scaled score: difference = −0.60; 95% CI: −1.16, −0.05 points) and Color-Word Interference (completion time scaled score: difference = −0.53; 95% CI: −1.08, 0.01 points) (Table 2). The adverse associations of Mn with Trail-Making and Color-Word Interference were supported by the BKMR results, where we observed decreasing Trail-Making and Color-Word Interference scaled scores as Mn levels increased, while the remaining chemicals were assigned their median values (Figure 1).

The associations of Mn with Verbal and Design Fluency were also negative, though weaker and with confidence intervals that crossed the null. We did not find evidence of any other consistent adverse associations between the remaining chemicals and cognitive flexibility outcomes. The BKMR results were similar with suggestive negative associations between certain exposures and cognitive flexibility (e.g., DDE and Trail-Making), however credible intervals were also wide (Figure 1). When we accounted for potential selection bias using IPW, the results were largely unchanged (Table S1).

### 3.6. Assessment of Effect Modification by Sex and PNSDI

In sex-stratified analyses, there was little evidence of differences in associations by sex (Table 3).

There was suggestive evidence of an adverse association of Mn with Trail-Making among females, but not males. However, this pattern of sex differences in associations was not observed for the other cognitive flexibility outcomes (Table 3). Unexpectedly, Pb was associated with better performance on the Verbal Fluency subtest in males, and HCB was associated with better performance on the Design Fluency subtest in females.

We found some evidence of potential interactions between PNSDI and the organochlorine chemicals but, for the most part, none of the stratum-specific estimates or their differences were statistically significant (Table 4).

Specifically, the association of HCB with Design Fluency differed between the two PNSDI groups. In addition, although the Mn-PNSDI interaction was not statistically significant, there was suggestive evidence of an adverse association between Mn and Trail-Making among those with a PNSDI < 3. The sex-stratified and PNSDI-stratified IPW results were similar to the complete case results (Tables S2 and S3).

### 3.7. Secondary Analyses: Negative Binomial and Logistic Regression Analyses of Cognitive Flexibility and Prenatal Chemical Mixture Exposures

When we analyzed the association between the five-chemical mixture and cognitive flexibility error raw scores, we found that a doubling of prenatal Mn concentrations was statistically significantly associated with an increased rate of Trail-Making errors (rate ratio = 1.30; 95% CI: 1.01, 1.67) and a doubling of prenatal DDE concentrations was statistically significantly associated with an increased rate of Verbal Fluency errors (rate ratio = 1.23; 95% CI: 1.05, 1.43) (Table S4). There was also suggestive evidence of an adverse association between Pb and Color-Word Interference total errors (rate ratio = 1.09; 95% CI: 0.99, 1.20). The remaining associations analyzed had wide confidence limits that crossed the null.

When we combined speed and accuracy to create outcomes representing overall Trail-Making and Color-Word Interference performance, we found evidence of a potential adverse association of Mn with Color-Word Interference (Table S5). Specifically, a twofold increase in Mn concentrations was associated with a 1.77 increased odds of being in the poor Color-Word Interference performance group (95% CI: 0.99, 3.10). In addition, we found evidence of adverse associations of ΣPCB_4_ and Pb with Color-Word Interference (ΣPCB_4_ OR = 1.54; 95% CI: 1.09, 2.17; Pb OR = 1.34; 95% CI: 0.98, 1.82). The IPW results of both the negative binomial regression and logistic regression analyses were similar to the complete case analyses (Tables S6 and S7).

### 3.8. Secondary Analyses: Seven-Chemical Mixture

In secondary analyses, we assessed the relationship between prenatal biomarkers of MeHg and As in addition to DDE, HCB, ΣPCB_4_, Pb, and Mn with cognitive flexibility among 235 NBC adolescents who had complete exposure, covariate, and outcome data. Chemical biomarker levels of MeHg in the NBC study participants were similar to those observed in high fish-eating populations [50] and As levels were similar to the general population [51] (Table S8). Participants who were included in the secondary analyses performed better on Trail-Making than those who were excluded, had higher cord serum DDE levels and lower cord blood Pb levels, and had characteristics consistent with greater sociodemographic advantage (Table S8). Among participants in the secondary analysis group, DDE and ΣPCB_4_ were strongly correlated with each other (Spearman *r* = 0.7), HCB was moderately correlated with DDE and ΣPCB_4_ (Spearman *r* = 0.4 for both correlations), and MeHg was moderately correlated with the organochlorines with the strongest correlations between MeHg and ΣPCB_4_ (Spearman *r* = 0.5) and MeHg and DDE (Spearman *r* = 0.4). The remaining metals were not well-correlated with each other or with the organochlorines (Spearman *r* = 0–0.2).

The results of exploratory BKMR analyses of the association between the seven-chemical mixture and cognitive flexibility scaled scores indicated potential non-linear relationships between Mn and Design Fluency as well as DDE and Color-Word Interference (Figure S1). Results of a likelihood ratio test comparing models with and without linear and quadratic terms for Mn (*p*-value = 0.008) and with and without linear and quadratic terms for DDE (*p*-value = 0.01) confirmed these non-linear associations. Therefore, we included a quadratic term for Mn in the Design Fluency model and a quadratic term for DDE in the Color-Word Interference model. We did not observe any interactions between the exposures in their association with the cognitive flexibility outcomes (Figure S2). When we used BKMR to assess the joint association of the chemicals on cognitive flexibility scaled scores, we found evidence of adverse joint associations of the seven-chemical mixture with both Trail-Making and Color-Word completion time scaled scores (Figure S3).

Similar to the main linear regression results, the secondary linear regression results provided some evidence of a negative association between Mn and cognitive flexibility scaled scores though with less precision due to a smaller sample size (Table S9). The adverse Mn-cognitive flexibility findings were supported by the BKMR results, particularly for the adverse association between Mn and Trail-Making (Figure S1). Mn appeared to have an upside-down U-shaped association with Design Fluency with a poorer performance at the lowest and highest concentrations of Mn (Figure S1). DDE appeared to have a U-shaped association with Color-Word Interference with better performance at the lowest and highest concentrations of DDE (Figure S1). We did not find evidence of adverse associations of MeHg or As with cognitive flexibility in either the linear regression or the BKMR analyses (Figure S1). We did observe a statistically significant adverse association between ΣPCB_4_ and Color-Word Interference that was stronger than what we had observed in the main analyses (completion time scaled score difference = −0.57; 95% CI: −1.02, −0.12 points as compared to −0.21 (−0.52, 0.10) points). However, this was likely due to population differences between those included in the main analyses (*n* = 373) versus those included in the secondary analyses (*n* = 235). For example, in secondary analyses restricted to *n* = 235, the ΣPCB_4_-Color-Word Interference association was similar whether or not MeHg and As were included in the model (data not shown). An adverse ΣPCB_4_-Color-Word Interference association was also observed in the BKMR analyses of the seven-chemical mixture (Figure S1). IPW results were similar to complete-case analyses (Table S10).

## 4. Discussion

In this study, we examined the hypothesized association of prenatal exposure to a five-chemical mixture composed of DDE, HCB, ΣPCB_4_, Pb, and Mn with cognitive flexibility among adolescents. Our analyses provided suggestive evidence that after adjusting for all our targeted neurotoxic exposures and covariates, cord blood Mn levels were adversely associated with cognitive flexibility among adolescents in the NBC. The same pattern of findings was demonstrated by the BKMR exposure-response functions that displayed individual chemical-cognitive flexibility associations within the context of a mixture (Figure 1). The completion time scaled scores for Trail-Making and Color-Word Interference tasks were most adversely impacted by Mn (Trail-Making completion time scaled score: difference = −0.60; 95% CI: −1.16, −0.05 points; Color-Word Interference completion time scaled score: difference = −0.53; 95% CI: −1.08, 0.01 points). Results were largely unchanged when we used IPW to account for potential selection bias due to loss to follow-up (Table S1). In addition, Mn was associated with an increased risk of committing Trail-Making errors (Table S4) and an increased odds of being in the worst performance group for Trail-Making (Table S5), using a derived outcome that accounted for both speed and accuracy. The adverse association between Mn and cognitive flexibility was still evident in the secondary analyses which included MeHg and As as part of the chemical mixture. Cognitive flexibility scaled scores are age-standardized to a mean of 10, SD of 3. Thus, our observed Mn-associated mean difference of 0.6 points for Trail-making represents 20% of an SD. This modest change may not have a clinical impact on the individual level. However, on the population level, small shifts in mean performance on this cognitive task may disproportionately impact the risk of impairment among those performing at the lower end of the distribution [52].

Mn is an essential trace element that is neurotoxic at higher levels [53]. As there is no identified industrial source of Mn exposure located near the NBC study communities, participants were likely exposed to Mn via multiple sources including diet, the most common source of Mn in the general population [54]. Although this is the first prospective study to analyze the association between prenatal Mn exposure and cognitive flexibility specifically, other studies have explored the relation of prenatal Mn with other executive functions such as inhibition and working memory. Within the NBC study, we previously found evidence of adverse associations of cord blood Mn with a verbal inhibition task measured by the D-KEFS Color-Word Interference inhibition condition [55], while a small exploratory study found that tooth Mn concentrations representative of prenatal exposure were associated with decrements in behavioral inhibition as measured by a Forbidden Toy Task, errors of commission on a Continuous Performance Task (CPT), and the Stroop Test among young children [22]. Prenatal Mn in the NBC was also associated with lower verbal and symbolic working memory scores measured by the Wide Range Assessment of Memory and Learning, 2nd Edition (WRAML2) [56] and tooth Mn concentrations were associated with decrements in working memory among adolescent girls, but not boys, living near ferromanganese industries in Italy [23].

We did not observe consistent patterns of adverse associations between the other individual chemicals (DDE, HCB, ΣPCB_4_, Pb) and cognitive flexibility outcomes in the standard linear regression, logistic regression, or negative binomial regression analyses considering all exposures in the same model (Table 2, Tables S4 and S5). Again, the same pattern of findings that was observed with standard multi-exposure linear regression, was also demonstrated by the BKMR exposure-response functions (Figure 1). These largely null associations may be due to lower exposure levels among NBC participants than those seen in other studies [47] or a limited sample size. In the case of the organochlorines that were assessed in this study, negative confounding by diet may have biased results [57]. In this study, a food frequency questionnaire was used to measure maternal seafood consumption during pregnancy, which may not fully capture dietary intake of seafood—a source of beneficial nutrients as well as PCB and MeHg exposures—resulting in some residual confounding by diet.

Using BKMR, we noted adverse joint associations of the five-chemical mixture with Trail-Making completion time and Color-Word completion time scaled scores (Figure 3). These joint associations were still present but somewhat attenuated when MeHg and As were included in the chemical mixture (Figure S3). This provides suggestive evidence that there may be an adverse relation between simultaneous exposure to these multiple chemicals and cognitive flexibility that is not apparent when assessing the impact of each exposure in multiple exposure parametric regression models. This combination of exposures has not been studied previously in relation to cognitive outcomes outside of the NBC. However, within the NBC, we previously observed adverse joint associations between these five chemicals and psychometric tests of inhibition and working memory [55,56].

We found some potential evidence of an interaction between HCB and sex in their relation to Design Fluency total correct scaled scores, however, the only previous study of HCB and executive function did not find any evidence of an HCB-sex interaction [20]. Whether age at assessment (age four compared to adolescents) or other factors may be important to potential sex differences in associations of HCB with executive function is uncertain and should be explored further. We did not observe statistically significant evidence of sex differences in associations between the remaining chemicals and cognitive flexibility outcomes.

We observed a statistically significant interaction between HCB and PNSDI in their association with Design Fluency wherein those who had a PNSDI <3 had a positive association. In PNSDI-stratified analyses, there was suggestive evidence of an adverse association between Mn and Trail-Making among those with a PNSDI <3 but the Mn and PNSDI interaction was not significant. The remaining interactions and stratum-specific associations were imprecise and therefore limit possible inferences. Although we did not observe significant interactions between PNSDI and the remaining chemicals, the HCB and Mn findings underscore the importance of examining potential effect modification by socio-demographic stressors. Similar patterns were observed in PNSDI-stratified analyses of the same chemical mixture with inhibition [55] and working memory [56] in the NBC cohort.

This study had some limitations including the loss to follow-up that may result in selection bias, limited data on MeHg and As exposures, potential residual confounding by diet or unmeasured factors, and issues regarding Mn measurement in cord blood. To account for loss to follow-up, we used IPW for censoring and found that in most cases, results were very similar to the complete-case results, meaning selection bias was unlikely to have impacted study findings. Because MeHg and As samples were collected two weeks post-partum at a home visit, rather than in the hospital after the child’s birth, we were not able to capture exposure among many participants resulting in potential loss of power to observe the effects of these exposures. Therefore, the relation between prenatal exposure to MeHg and As should be explored further in larger samples. As previously mentioned, although we were able to account for maternal seafood consumption during pregnancy, this information was obtained with a food frequency questionnaire which may not fully capture diet. This could result in residual negative confounding by seafood consumption and an underestimation of the adverse association between certain chemicals such as the organochlorines (exposure to which is associated with seafood consumption) and cognitive flexibility. Two limitations in using cord blood Mn as a biomarker of Mn exposure include a lack of consensus about which biological matrix is the most valid biomarker of prenatal Mn exposure and using ICP-MS to detect cord blood Mn as the Mn isotope has a similar mass to two isotopes of iron which may result in iron contributing to the Mn signal among those with high iron levels [58]. However, there is evidence that cord blood Mn correlates well with third-trimester tooth Mn and is, therefore, a valid biomarker of prenatal Mn exposure [59,60,61] and adequate separation of Mn and iron occurred in the laboratory in which these analyses took place.

This study also had important strengths including a socio-demographically diverse study population, a prospective study design, biomarkers of prenatal exposure to multiple organochlorines and metals, comprehensive psychometric measures of cognitive flexibility in adolescence (an age group for whom manifestations of adverse executive function development may be most evident), and detailed sociodemographic, dietary, and lifestyle information. These factors allowed us to conduct an in-depth investigation of the association of prenatal exposure to a chemical mixture composed of neurotoxic organochlorines and metals with cognitive flexibility among adolescents while adjusting for important potential confounders and exploring effect modifiers, including indicators of social disadvantage. Using parametric regression analyses, we found suggestive evidence of an adverse impact of prenatal Mn on adolescent cognitive flexibility, while accounting for other chemicals and covariates. Using BKMR, we also observed an adverse joint association between the full chemical mixture on multiple measures of cognitive flexibility. Finally, in some analyses, we observed the potential for stronger adverse associations between prenatal exposures to organochlorine pesticides and cognitive flexibility outcomes among participants with more prenatal social disadvantage, highlighting the potential for exposure to psychosocial stressors to enhance susceptibility to prenatal chemical exposures. The study findings contribute to the limited literature assessing the relation of chemical mixtures and executive function among adolescents while underscoring the importance of analyzing the impact of multiple exposures simultaneously and focusing on high-risk populations.

## Figures and Tables

**Table 1:** Characteristics of New Bedford Cohort participants who were included in the main analysis group ^1^ and those who were excluded from the main analysis.

Descriptive Characteristic	Main Analysis Group, *n* = 373			Excluded Group, *n* = 415			
Cognitive Flexibility Measures ^2^	*n* (%)	Mean (SD)	Range	*n* (%)	Mean (SD)	Range	*p*-Value ^3^
Trail-Making							
Completion time scaled score	373	9.6 (2.8)	1–14	155	9.1 (2.8)	1–14	0.1
Total errors	373	0.9 (1.1)	0–5	154	1.0 (1.4)	0–13	0.5
Overall Trail-Making performance							
Best performance	113 (30.3)			45 (10.8)			0.9
Poor performance	260 (69.7)			109 (26.3)			
Missing	0			261 (62.9)			
Verbal Fluency							
Switching accuracy scaled score	373	9.2 (2.8)	2–17	155	9.0 (2.8)	1–17	0.3
Total errors	373	0.8 (1.2)	0–7	155	0.9 (1.3)	0–7	0.4
Design Fluency							
Total correct scaled score	373	9.9 (2.8)	2–18	155	9.6 (2.6)	2–17	0.3
Total errors	373	2.6 (3.1)	0–22	155	2.6 (2.6)	0–16	0.2
Color-Word Interference							
Completion time scaled score	373	9.9 (2.6)	1–15	154	9.8 (2.7)	1–14	0.7
Total errors	373	2.6 (2.4)	0–19	154	2.8 (2.4)	0–11	0.4
Overall Color-Word performance							
Best performance	83 (22.3)			34 (8.2)			1.0
Poor performance	290 (77.7)			120 (28.9)			
Missing	0			261 (62.9)			
**Exposure Measures ^4^**							
Cord serum DDE (ng/g)	373	0.6 (1.2)	0.02–14.9	378	0.4 (0.4)	0.0–4.2	<0.01 *
Cord serum HCB (ng/g)	373	0.03 (0.02)	0.0–0.1	378	0.03 (0.05)	0.0–0.7	0.1
Cord serum ΣPCB_4_ (ng/g)	373	0.3 (0.3)	0.01–4.4	378	0.2 (0.2)	0.01–1.9	0.05
Cord blood Pb (μg/dL)	373	1.4 (0.9)	0.0–9.4	375	1.7 (1.7)	0.0–17.4	<0.01 *
Cord blood Mn (µg/dL)	373	4.2 (1.6)	0.7–14.6	335	4.3 (2.0)	0.2–22.1	0.6
**Covariate Measures ^5^**							
Child Characteristics							
Race/Ethnicity							0.09
Non–Hispanic White	263 (70.5)			268 (64.6)			
Hispanic	33 (8.8)			56 (13.5)			
Other	77 (20.6)			89 (21.4)			
Missing	0			2 (0.5)			
Sex							0.05
Male	179 (48.0)			229 (55.2)			
Female	194 (52.0)			186 (44.8)			
Age at Exam	373	15.5 (0.6)	14.4–17.8	155	15.7 (0.7)	13.9–17.9	<0.01 *
Home Score	373	43.9 (6.3)	21–56	118	42.7 (6.0)	27–53	0.07
Year of Birth							<0.01 *
1993–1994	100 (26.8)			159 (38.3)			
1995–1996	153 (41.0)			147 (35.4)			
1997–1998	120 (32.2)			109 (26.3)			
Maternal Characteristics							
Marital status at birth							<0.01 *
Not married	136 (36.5)			195 (47.0)			
Married	237 (63.5)			165 (39.8)			
Missing	0			55 (13.3)			
Maternal IQ	373	99.4 (10.4)	57–124	262	95.8 (10.2)	72–126	<0.01 *
Seafood during pregnancy (serv/day)	373	0.5 (0.6)	0–5.3	260	0.6 (0.7)	0–6	0.6
Smoking during pregnancy							0.1
No	272 (72.9)			210 (50.6)			
Yes	101 (27.1)			103 (24.8)			
Missing	0			102 (24.6)			
Household Characteristics at Birth							
Maternal education							<0.01 *
≤High School	190 (50.9)			231 (55.7)			
>High School	183 (49.1)			127 (30.6)			
Missing	0			57 (13.7)			
Paternal Education							<0.01 *
≤High School	246 (66.0)			266 (64.1)			
>High School	127 (34.0)			81 (19.5)			
Missing	0			68 (16.4)			
Annual Household Income							<0.01 *
<$20,000	115 (30.8)			150 (36.1)			
≥$20,000	258 (69.2)			201 (48.4)			
Missing	0			64 (15.4)			
Examination Characteristics							
Examiner							0.4
1	277 (74.3)			121 (29.2)			
2	96 (25.7)			34 (8.2)			
Missing	0			260 (62.7)			

^1^ Main analysis group: complete outcome, covariate and prenatal exposure biomarker data for DDE, HCB, ΣPCB_4_, Pb and Mn, *n* = 373. ^2^ NBC participants with missing cognitive flexibility measures: Trail-Making completion time *n* = 260; Trail-Making total errors *n* = 261; Verbal Fluency switching accuracy *n* = 260; Verbal Fluency total errors *n* = 260; Design Fluency total correct *n* = 260; Design Fluency total errors *n* = 260; Color-Word Interference completion time *n* = 261; Color-Word Interference total errors *n* = 261. ^3^ *p*-values represent results comparing characteristics between participants included in the main analysis and those excluded from the main analysis using *t*-tests, chi-square tests, and Wilcoxon rank sum tests where appropriate. *p*-values reflect comparisons between groups with non-missing data. ^4^ NBC participants with missing exposure measures: DDE *n* = 37; HCB *n* = 37; ΣPCB_4_ *n* = 37; Pb *n* = 40; Mn *n* = 80. ^5^ NBC participants with missing covariate measures: age at exam *n* = 260; HOME score *n* = 297; maternal IQ *n* = 153; seafood during pregnancy *n* = 155. * *p* < 0.05. Abbreviations: DDE: dichlorodiphenyldichloroethylene; HCB: hexachlorobenzene; ΣPCB_4_: Sum of 4 PCB congeners (118, 138, 153, 180); Pb: lead; Mn: manganese.

**Table 2:** Complete-case results of multivariable linear regression analyses (difference in scaled scores associated with a twofold increase in exposure and 95% CI) ^1^ assessing the relation of prenatal exposure to a five-chemical mixture with Delis-Kaplan Executive Function System (D-KEFS) cognitive flexibility scaled scores among adolescents in the main analysis group ^2^.

Exposure	Trail-Making Completion Time Difference (95% CI)	Verbal Fluency Switching Accuracy Difference (95% CI)	Design Fluency Total Correct Difference (95% CI)	Color-Word Interference Completion Time Difference (95% CI)
Log_2_ DDE	−0.23 (−0.52, 0.06)	−0.10 (−0.40, 0.20)	−0.01 (−0.32, 0.30)	0.06 (−0.22, 0.34)
Log_2_ HCB	0.11 (−0.20, 0.42)	−0.06 (−0.38, 0.26)	0.24 (−0.09, 0.58)	0.05 (−0.26, 0.35)
Log_2_ ΣPCB_4_	0.02 (−0.30, 0.34)	0.05 (−0.27, 0.38)	−0.09 (−0.44, 0.25)	−0.21 (−0.52, 0.10)
Log_2_ Pb	0.14 (−0.16, 0.43)	0.27 (−0.03, 0.57)	0.05 (−0.27, 0.36)	0.04 (−0.24, 0.33)
Log_2_ Mn	−0.60 (−1.16, −0.05) *	−0.28 (−0.85, 0.29)	−0.10 (−0.70, 0.50)	−0.53 (−1.08, 0.01)

^1^ Exposures have been log_2_-transformed and models have been adjusted for child race, sex, age at exam, year of birth, and HOME score; maternal marital status at child’s birth, IQ, seafood consumption during pregnancy, and smoking during pregnancy; maternal and paternal education and annual household income at child’s birth; and study examiner. ^2^ Main analysis group: complete outcome, covariate, and prenatal exposure biomarker data for DDE, HCB, ΣPCB_4_, Pb and Mn, *n* = 373. * *p* < 0.05. Abbreviations: DDE: dichlorodiphenyldichloroethylene; HCB: hexachlorobenzene; ΣPCB_4_: Sum of 4 PCB congeners (118, 138, 153, 180); Pb: lead; Mn: manganese.

**Table 3:** Sex-stratified complete-case results of multivariable linear regression analyses (difference in scaled scores associated with a twofold increase in exposure and 95% CI) ^1^ assessing the relation of prenatal exposure to a five-chemical mixture with Delis-Kaplan Executive Function System (D-KEFS) cognitive flexibility scaled scores among adolescents in the main analysis group ^2^.

**Exposure**	**Trail-Making Completion Time**			**Verbal Fluency Switching Accuracy**		
	**Difference (95% CI)**			**Difference (95% CI)**		
	**Males**	**Females**	***p* for Interaction**	**Males**	**Females**	***p* for Interaction**
Log2 DDE	−0.31 (−0.73, 0.12)	−0.14 (−0.58, 0.30)	0.6	−0.24 (−0.65, 0.16)	0.02 (−0.45, 0.50)	0.3
Log2 HCB	0.09 (−0.37, 0.55)	0.14 (−0.31, 0.59)	0.6	−0.22 (−0.67, 0.22)	−0.07 (−0.56, 0.42)	0.9
Log2 ΣPCB4	0.18 (−0.29, 0.65)	−0.16 (−0.63, 0.32)	0.6	0.10 (−0.35, 0.55)	−0.04 (−0.55, 0.48)	0.4
Log2 Pb	0.44 (−0.10, 0.97)	−0.01 (−0.37, 0.35)	0.1	0.64 (0.12, 1.15) *	0.09 (−0.30, 0.48)	0.2
Log2 Mn	−0.15 (−0.95, 0.66)	−0.80 (−1.59, 0.00)	0.3	−0.47 (−1.25, 0.31)	−0.05 (−0.91, 0.81)	0.6
**Exposure**	**Design Fluency Total Correct Difference (95% CI)**			**Color-Word Interference Completion Time**		
				**Difference (95% CI)**		
	**Males**	**Females**	***p* for Interaction**	**Males**	**Females**	***p* for Interaction**
Log2 DDE	0.20 (−0.27, 0.67)	−0.10 (−0.57, 0.36)	0.4	0.09 (−0.34, 0.52)	0.10 (−0.31, 0.51)	0.9
Log2 HCB	−0.15 (−0.67, 0.36)	0.73 (0.26, 1.20) *	0.01 *	−0.02 (−0.49, 0.44)	0.07 (−0.34, 0.49)	0.6
Log2 ΣPCB4	−0.04 (−0.56, 0.48)	−0.29 (−0.79, 0.21)	0.7	−0.37 (−0.84, 0.10)	−0.08 (−0.53, 0.36)	0.2
Log2 Pb	0.29 (−0.31, 0.88)	−0.10 (−0.47, 0.28)	0.2	0.03 (−0.51, 0.57)	−0.03 (−0.36, 0.31)	0.8
Log2 Mn	−0.45 (−1.35, 0.44)	0.40 (−0.43, 1.23)	0.2	−0.54 (−1.35, 0.27)	−0.31 (−1.05, 0.43)	0.8

^1^ Exposures have been log_2_-transformed and models have been adjusted for child race, sex, age at exam, year of birth, and HOME score; maternal marital status at child’s birth, IQ, seafood consumption during pregnancy, and smoking during pregnancy; maternal and paternal education and annual household income at child’s birth; and study examiner. ^2^ Main analysis group: complete outcome, covariate, and prenatal exposure biomarker data for DDE, HCB, ΣPCB_4,_ Pb and Mn. Total *n* = 373; Males *n* = 179; Females *n* = 194. * *p* < 0.05. Abbreviations: DDE: dichlorodiphenyldichloroethylene; HCB: hexachlorobenzene; ΣPCB_4_: Sum of 4 PCB congeners (118, 138, 153, 180); Pb: lead; Mn: manganese.

**Table 4:** Prenatal social disadvantage index (PNSDI) ^1^ stratified complete-case results of multivariable linear regression analyses (difference in scaled scores associated with a twofold increase in exposure and 95% CI) ^2^ assessing the relation of prenatal exposure to a five-chemical mixture with Delis-Kaplan Executive Function System (D-KEFS) cognitive flexibility scaled scores among adolescents in the main analysis group ^3^.

**Exposure**	**Trail-Making Completion Time**			**Verbal Fluency Switching Accuracy**		
	**Difference (95% CI)**			**Difference (95% CI)**		
	**PNSDI < 3**	**PNSDI ≥ 3**	***p* for Interaction**	**PNSDI < 3**	**PNSDI ≥ 3**	***p* for Interaction**
Log_2_ DDE	−0.13 (−0.46, 0.20)	−0.36 (−0.97, 0.25)	0.3	−0.12 (−0.48, 0.25)	−0.15 (−0.71, 0.41)	0.9
Log_2_ HCB	0.23 (−0.14, 0.60)	−0.22 (−0.82, 0.38)	0.1	0.06 (−0.35, 0.46)	−0.36 (−0.92, 0.19)	0.2
Log_2_ ΣPCB_4_	−0.10 (−0.47, 0.26)	0.42 (−0.25, 1.09)	0.2	0.10 (−0.30, 0.50)	0.16 (−0.46, 0.78)	0.9
Log_2_ Pb	−0.01 (−0.37, 0.35)	0.09 (−0.49, 0.67)	0.7	0.20 (−0.19, 0.60)	0.26 (−0.27, 0.80)	0.6
Log_2_ Mn	−0.65 (−1.31, 0.02)	−0.37 (−1.44, 0.70)	0.8	−0.27 (−1.00, 0.45)	−0.22 (−1.21, 0.77)	0.7
**Exposure**	**Design Fluency Total Correct Difference (95% CI)**			**Color-Word Interference Completion Time**		
				**Difference (95% CI)**		
	**PNSDI < 3**	**PNSDI ≥ 3**	***p* for interaction**	**PNSDI < 3**	**PNSDI ≥ 3**	***p* for Interaction**
Log_2_ DDE	0.05 (−0.35, 0.45)	−0.18 (−0.71, 0.35)	0.4	0.20 (−0.12, 0.53)	−0.08 (−0.65, 0.49)	0.2
Log_2_ HCB	0.59 (0.15, 1.04) *	−0.38 (−0.90, 0.14)	0.01 *	0.13 (−0.23, 0.50)	−0.12 (−0.68, 0.44)	0.3
Log_2_ ΣPCB_4_	−0.29 (−0.73, 0.15)	0.42 (−0.17, 1.00)	0.1	−0.35 (−0.71, 0.00)	0.21 (−0.41, 0.84)	0.2
Log_2_ Pb	−0.07 (−0.51, 0.36)	0.12 (−0.38, 0.62)	0.5	−0.18 (−0.54, 0.18)	0.20 (−0.34, 0.74)	0.3
Log_2_ Mn	−0.12 (−0.92, 0.68)	−0.26 (−1.19, 0.67)	0.8	−0.50 (−1.16, 0.15)	−0.50 (−1.51, 0.50)	0.9

^1^ Prenatal social disadvantage index (PNSDI) was constructed as the sum of five adverse social or economic exposures at the time of the child’s birth where the presence of each risk factor was assigned a value of 1, absence a value of 0: mother unmarried, mother’s education as a high school graduate or less, father’s education as a high school graduate or less, annual household income less than $20,000, and mother’s age at birth less than 20 years.^2^ Exposures have been log_2_-transformed and models have been adjusted for child race, sex, age at exam, year of birth, and HOME score; maternal marital status at child’s birth, IQ, seafood consumption during pregnancy, and smoking during pregnancy; maternal and paternal education and annual household income at child’s birth; and study examiner. ^3^ Main analysis group: complete outcome, covariate, and prenatal exposure biomarker data for DDE, HCB, ΣPCB_4,_ Pb, and Mn. Total *n* = 373; PNSDI < 3, *n* = 241; PNSDI ≥ 3, *n* = 132. * *p* < 0.05. Abbreviations: DDE: dichlorodiphenyldichloroethylene; HCB: hexachlorobenzene; ΣPCB_4_: Sum of 4 PCB congeners (118, 138, 153, 180); Pb: lead; Mn: manganese.

## Data Availability

The data are not publicly available due to privacy and confidentiality reasons.

## Supplementary Materials

The following are available online at https://www.mdpi.com/article/10.3390/toxics9120329/s1, Figure S1: Estimated covariate-adjusted exposure-response functions and 95% credible intervals between DDE, HCB, ΣPCB_4_, Pb, Mn, MeHg, and As and Delis Kaplan Executive Function System (D-KEFS) cognitive flexibility scaled scores, among adolescents in the secondary analysis group. In each plot, all of the remaining exposures are assigned to their median value, Figure S2: Covariate-adjusted exposure-response functions between one of 7 exposures (DDE, HCB, ΣPCB_4_, Pb, Mn, MeHg, As) where a second exposure is fixed at various quantiles and Delis-Kaplan Executive Function System (D-KEFS) cognitive flexibility scaled scores, among adolescents in the secondary analysis group. In each plot, all of the remaining exposures are assigned their median value, Figure S3: Joint association between the chemical mixture composed of DDE, HCB, ΣPCB_4_, Pb, Mn, MeHg, and As (estimates and 95% credible intervals) and the Delis-Kaplan Executive Function System (D-KEFS) cognitive flexibility scaled scores: (**a**) Trail-Making completion time, (**b**) Verbal Fluency switching accuracy, (**c**) Design Fluency total correct, (**d**) Color-Word Interference completion time. Chemical mixture levels at various percentiles are compared to a mixture with each component at its median level, among New Bedford Cohort adolescents in the secondary analysis group, Table S1: Inverse-probability weighted results of multivariable linear regression analyses (difference in scaled scores associated with a twofold increase in exposure and 95% CI) assessing the relation of prenatal exposure to a five-chemical mixture with Delis-Kaplan Executive Function System (D-KEFS) cognitive flexibility scaled scores among adolescents in the main analysis group, Table S2: Sex-stratified inverse probability weighted results of multivariable linear regression analyses (difference in scaled scores associated with a twofold increase in exposure and 95% CI) assessing the relation of prenatal exposure to a five-chemical mixture with Delis-Kaplan Executive Function System (D-KEFS) cognitive flexibility scaled scores among adolescents in the main analysis group, Table S3: Prenatal social disadvantage index (PNSDI)-stratified inverse probability weighted results of multivariable linear regression analyses (difference in scaled scores associated with a twofold increase in exposure and 95% CI) assessing the relation of prenatal exposure to a five-chemical mixture with Delis-Kaplan Executive Function System (D-KEFS) cognitive flexibility scaled scores among adolescents in the main analysis group, Table S4: Complete-case results of negative binomial regression analyses (rate ratio (RR) and 95% CI) assessing the relation of prenatal exposure to a five- chemical mixture with Delis-Kaplan Executive Function System (D-KEFS) cognitive flexibility error raw scores among adolescents in the main analysis group, Table S5: Complete-case results of logistic regression analyses (odds ratio (OR) and 95% CI) assessing the relation of prenatal exposure to a five-chemical mixture with odds of poor performance on Delis-Kaplan Executive Function System (D-KEFS) overall Trail-Making and Color-Word Interference performance among adolescents in the main analysis group, Table S6: Inverse probability weighted results of negative binomial regression analyses (RR (rate ratio) and 95% CI) assessing the relation of prenatal exposure to a five-chemical mixture with Delis-Kaplan Executive Function System (D-KEFS) cognitive flexibility error raw scores among adolescents in the main analysis group, Table S7: Inverse probability weighted results of logistic regression analyses (odds ratio (OR) and 95% CI) assessing the relation of prenatal exposure to a five-chemical mixture with odds of poor performance on Delis-Kaplan Executive Function System (D-KEFS) overall Trail-Making and Color-Word Interference performance among adolescents in the main analysis group, Table S8: Characteristics of New Bedford Cohort participants who were included in the secondary analysis group, and those who were excluded from the secondary analysis group, Table S9: Complete-case results of multivariable linear regression analyses (difference in scaled scores associated with a twofold increase in exposure and 95% CI) assessing the relation of prenatal exposure to a seven-chemical mixture with Delis-Kaplan Executive Function System (D-KEFS) cognitive flexibility scaled scores among adolescents in the secondary analysis group, Table S10: Inverse-probability weighted results of multivariable linear regression analyses (difference in scaled scores associated with a twofold increase in exposure and 95% CI) assessing the relation of prenatal exposure to a seven-chemical mixture with Delis-Kaplan Executive Function System (D-KEFS) cognitive flexibility scaled scores among adolescents in the secondary analysis group.
