# Supplementary material for: Prenatal Exposure to Chemical Mixtures and Cognitive Flexibility among Adolescents
Source: Toxics. 2021 Dec 2;9(12):329. doi: 10.3390/toxics9120329 (PMC8708222; doi:10.3390/toxics9120329)
Supplement: Supplementary file 1 [file toxics-09-00329-s001.zip › toxics-1437846-supplementary.pdf]

toxics

Supplementary Materials: Prenatal Exposure to Chemical Mixtures and Cognitive Flexibility among Adolescents

Anna V. Oppenheimer, David C. Bellinger, Brent A. Coull, Marc G. Weisskopf and Susan A. Korrick

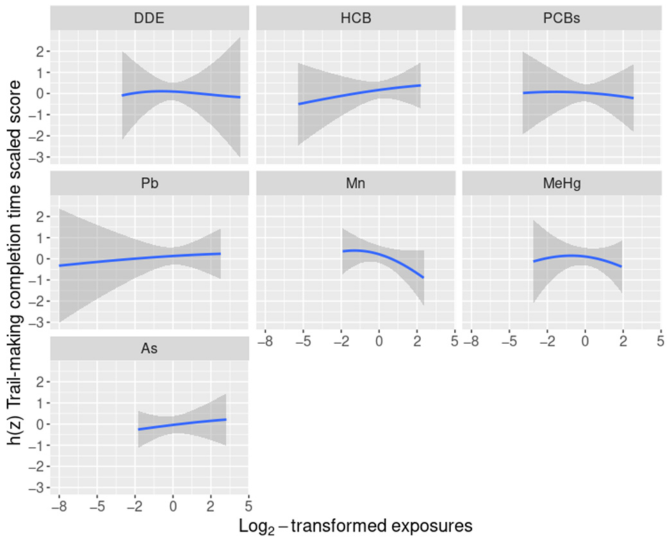

(a) Trail-Making completion time

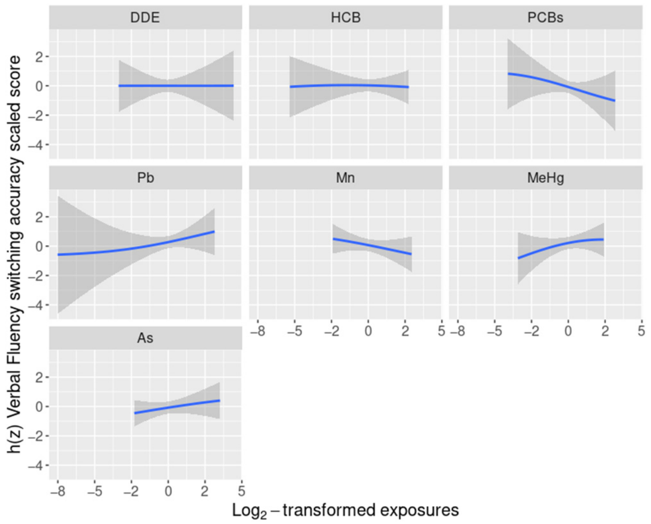

(b) Verbal Fluency switching accuracy

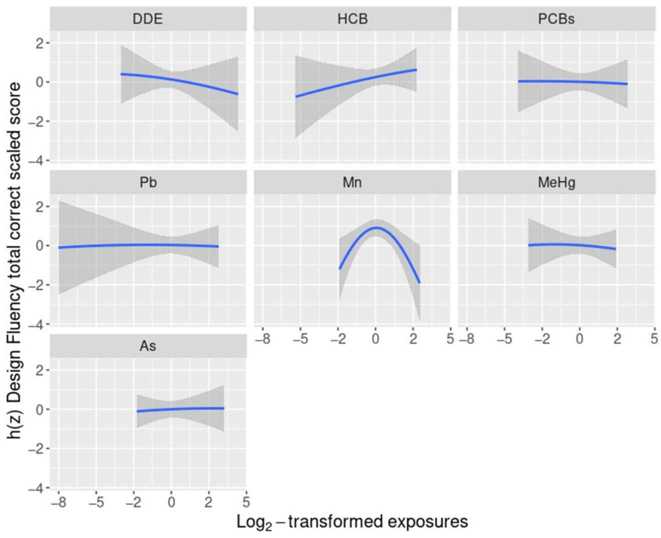

(c) Design Fluency total correct

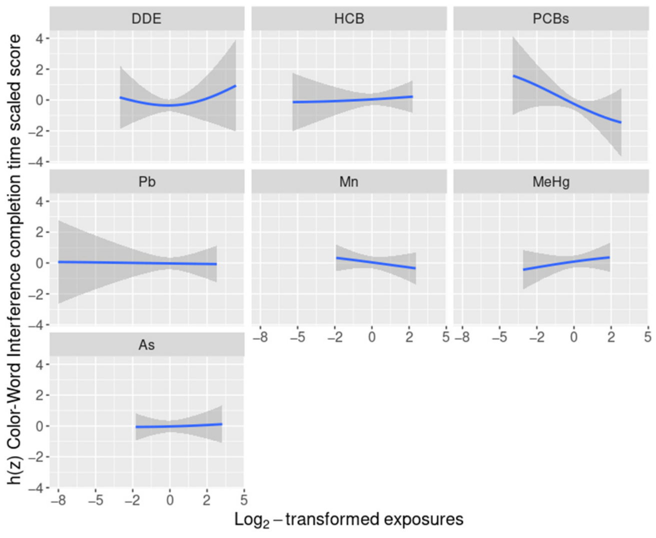

(d) Color-Word Interference completion time

Figure S1. Estimated covariate-adjusted exposure-response functions and 95% credible intervals[1] between DDE, HCB, ΣPCB_4, Pb, Mn, MeHg, and As and Delis Kaplan Executive Function System (D-KEFS) cognitive flexibility scaled scores: (**a**) Trail-Making completion time, (**b**) Verbal Fluency switching accuracy, (**c**) Design Fluency total correct, (**d**) Color-Word Interference completion time, among New Bedford Cohort adolescents in the secondary analysis group[2]. In each plot, all of the remaining exposures are assigned to their median value.

Footnotes: [1]Exposures have been log2-transformed and models have been adjusted for child race, sex, age at exam, year of birth, and HOME score; maternal marital status at child's birth, IQ, seafood consumption during pregnancy, and smok-

ing during pregnancy; maternal and paternal education and annual household income at child's birth; and study examiner. [2]Secondary analysis group: complete outcome, covariate and prenatal exposure biomarker data for DDE, HCB, ΣPCB_4, Pb, Mn, MeHg, and As, $n = 235$. Abbreviations: DDE: dichlorodiphenyldichloroethylene; HCB: hexachlorobenzene; ΣPCB_4: Sum of 4 PCB congeners (118, 138, 153, 180); Pb: lead; Mn: manganese; MeHg: methylmercury; As: arsenic.

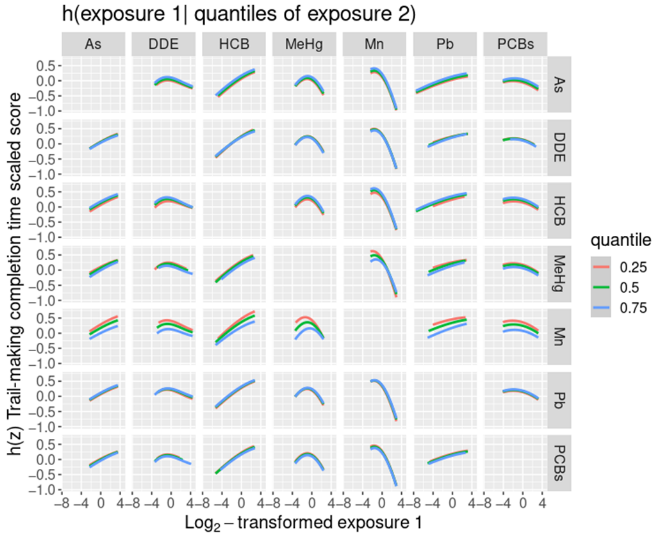

(a) Trail-Making completion time

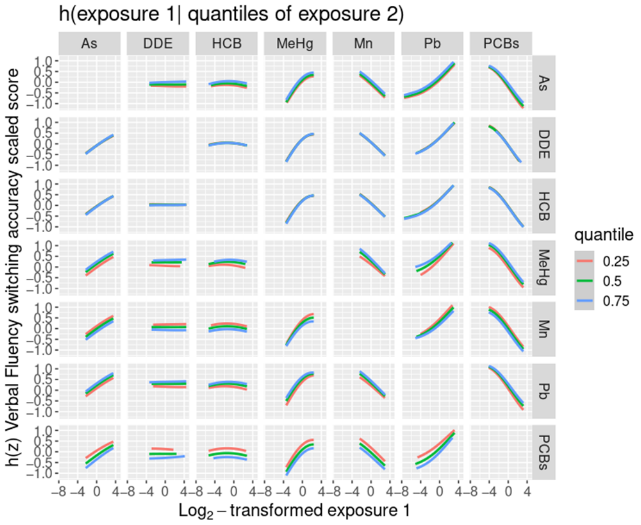

(b) Verbal Fluency switching accuracy

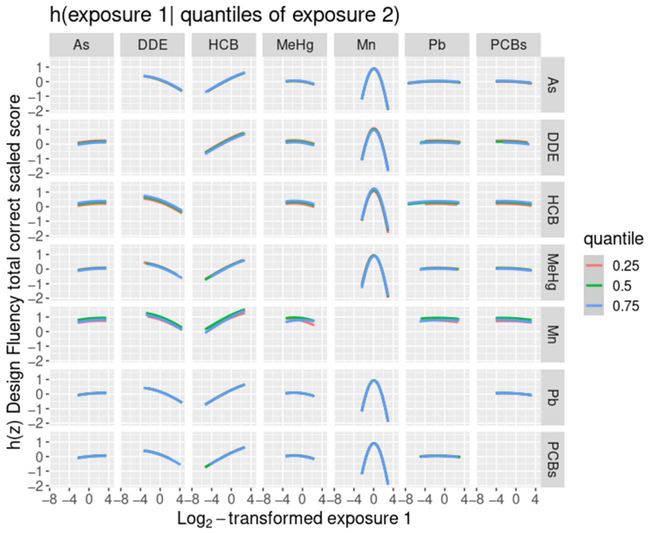

(c) Design Fluency total correct

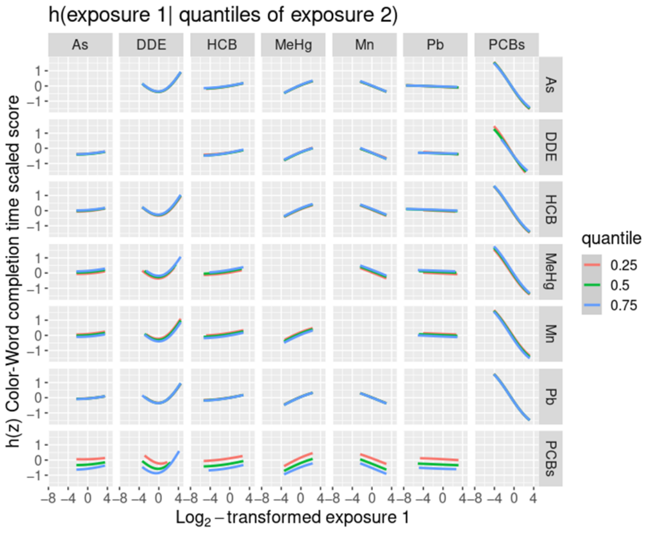

(d) Color-Word Interference completion time

Figure S2. Covariate-adjusted exposure-response functions[1] between one of 7 exposures (DDE, HCB, ΣPCB_4, Pb, Mn, MeHg, As) where a second exposure is fixed at various quantiles and Delis-Kaplan Executive Function System (D-KEFS) cognitive flexibility scaled scores: (**a**) Trail-Making completion time, (**b**) Verbal Fluency switching accuracy, (**c**) Design Fluency total correct, (**d**) Color-Word Interference completion time, among New Bedford cohort adolescents in the secondary analysis group[2]. In each plot, all of the remaining exposures are assigned their median value.

Footnotes: [1]Exposures have been log2-transformed and models have been adjusted for child race, sex, age at exam, year of birth, and HOME score; maternal marital status at child's birth, IQ, seafood consumption during pregnancy, and smoking during pregnancy; maternal and paternal education and annual household income at child's birth; and study examiner. [2]Secondary analysis group: complete outcome, covariate and prenatal exposure biomarker data for DDE, HCB, ΣPCB_4, Pb, Mn, MeHg, and As, $n = 235$. Abbreviations: DDE: dichlorodiphenyldichloroethylene; HCB: hexachlorobenzene; ΣPCB_4: Sum of 4 PCB congeners (118, 138, 153, 180); Pb: lead; Mn: manganese; MeHg: methylmercury; As: arsenic.

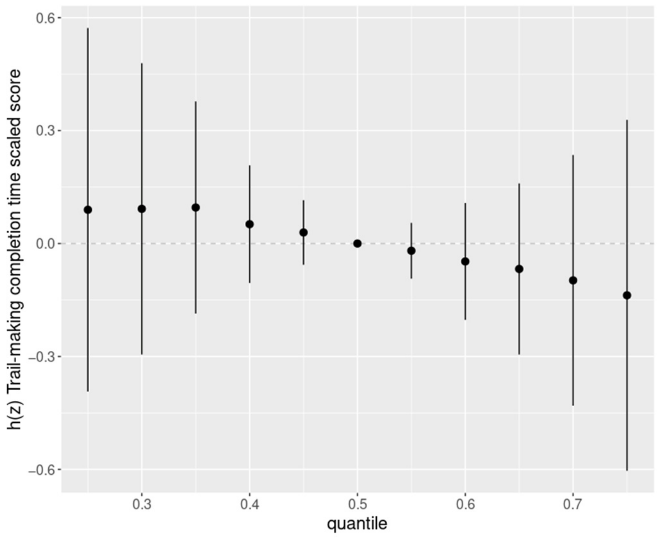

(a) Trail-Making completion time

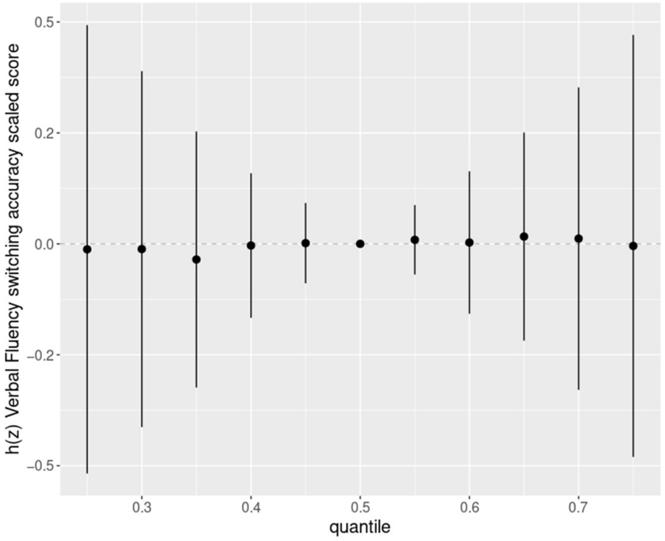

(b) Verbal Fluency switching accuracy

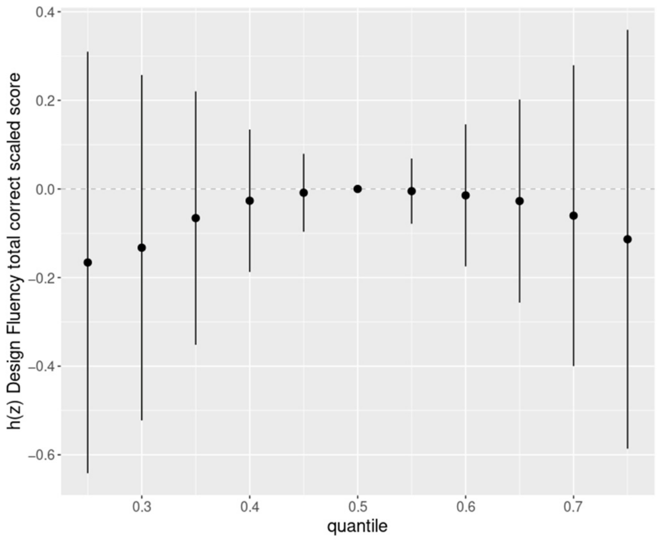

(c) Design Fluency total correct

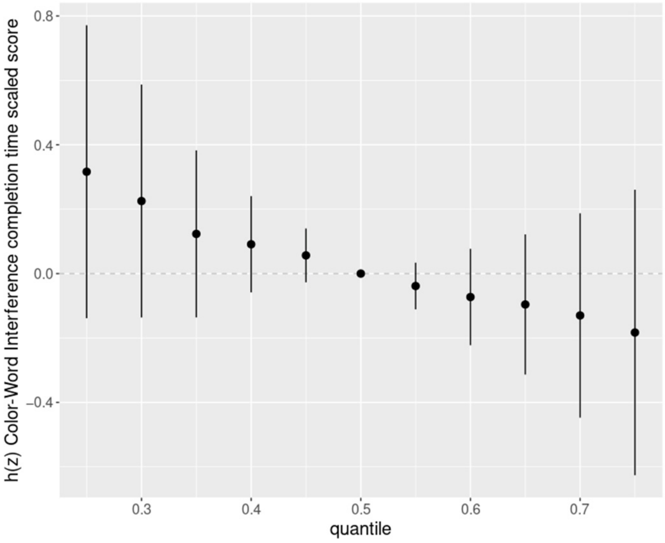

(d) Color-Word Interference completion time

Figure S3. Joint association between the chemical mixture composed of DDE, HCB, ΣPCB_4, Pb, Mn, MeHg, and As (estimates and 95% credible intervals[1]) and the Delis-Kaplan Executive Function System (D-KEFS) cognitive flexibility scaled scores: (**a**) Trail-Making completion time, (**b**) Verbal Fluency switching accuracy, (**c**) Design Fluency total correct, (**d**) Color-Word Interference completion time. Chemical mixture levels at various percentiles are compared to a mixture with each component at its median level, among New Bedford Cohort adolescents in the secondary analysis group[2].

Footnotes: [1]Exposures have been log2-transformed and models have been adjusted for child race, sex, age at exam, year of birth, and HOME score; maternal marital status at child's birth, IQ, seafood consumption during pregnancy, and smoking during pregnancy; maternal and paternal education and annual household income at child's birth; and study examiner. [2]Secondary analysis group: complete outcome, covariate and prenatal exposure biomarker data for DDE, HCB, ΣPCB_4, Pb, Mn, MeHg, and As, *n* = 235. Abbreviations: DDE: dichlorodiphenyldichloroethylene; HCB: hexachlorobenzene; ΣPCB_4: Sum of 4 PCB congeners (118, 138, 153, 180); Pb: lead; Mn: manganese; MeHg: methylmercury; As: arsenic.

Table S1. Inverse-probability weighted results of multivariable linear regression analyses (difference in scaled scores associated with a twofold increase in exposure and 95% CI)[1] assessing the relation of prenatal exposure to a five-chemical mixture with Delis-Kaplan Executive Function System (D-KEFS) cognitive flexibility scaled scores among New Bedford Cohort adolescents in the main analysis group[2].

Exposure	Trail-Making completion time	Verbal Fluency switching accuracy	Design Fluency total correct	Color-Word Interference completion time
	**Difference (95% CI)**	**Difference (95% CI)**	**Difference (95% CI)**	**Difference (95% CI)**
Log_2 DDE	−0.23 (−0.52, 0.06)	−0.08 (−0.37, 0.21)	0.02 (−0.29, 0.33)	0.04 (−0.25, 0.32)
Log_2 HCB	0.08 (−0.24, 0.39)	−0.10 (−0.42, 0.22)	0.14 (−0.19, 0.48)	0.03 (−0.28, 0.33)
Log_2 ΣPCB_4	0.07 (−0.25, 0.39)	0.03 (−0.29, 0.35)	−0.08 (−0.41, 0.26)	−0.17 (−0.48, 0.14)
Log_2 Pb	0.16 (−0.14, 0.46)	0.33 (0.02, 0.63) *	0.09 (−0.23, 0.41)	0.08 (−0.21, 0.38)
Log_2 Mn	−0.61 (−1.17, −0.04) *	−0.30 (−0.86, 0.27)	−0.19 (−0.78, 0.41)	−0.60 (−1.14, −0.05) *

[1]Exposures have been log2-transformed and models have been adjusted for child race, sex, age at exam, year of birth, and HOME score; maternal marital status at child's birth, IQ, seafood consumption during pregnancy, and smoking during pregnancy; maternal and paternal education and annual household income at child's birth; and study examiner. [2]Main analysis group: complete outcome, covariate and prenatal exposure biomarker data for DDE, HCB, ΣPCB_4, Pb and Mn, $n = 373$. *$p < 0.05$. Abbreviations: DDE: dichlorodiphenyldichloroethylene; HCB: hexachlorobenzene; ΣPCB_4: Sum of 4 PCB congeners (118, 138, 153, 180); Pb: lead; Mn: manganese.

Table S2. Sex-stratified inverse probability weighted results of multivariable linear regression analyses (difference in scaled scores associated with a twofold increase in exposure and 95% CI)[1] assessing the relation of prenatal exposure to a five-chemical mixture with Delis-Kaplan Executive Function System (D-KEFS) cognitive flexibility scaled scores among New Bedford Cohort adolescents in the main analysis group[2].

Exposure	Trail-Making completion time Difference (95 % CI)			Verbal Fluency switching accuracy Difference (95% CI)		
	Males	Females	*p* for interaction	Males	Females	*p* for interaction
Log_2 DDE	−0.29 (−0.71, 0.14)	−0.16 (−0.61, 0.28)	0.6	−0.24 (−0.65, 0.16)	0.08 (−0.40, 0.55)	0.2
Log_2 HCB	0.05 (−0.41, 0.52)	0.12 (−0.34, 0.57)	0.6	−0.28 (−0.72, 0.17)	−0.10 (−0.59, 0.39)	0.8
Log_2 ΣPCB_4	0.19 (−0.28, 0.66)	−0.09 (−0.57, 0.38)	0.7	0.10 (−0.35, 0.55)	−0.09 (−0.60, 0.42)	0.3
Log_2 Pb	0.47 (−0.07, 1.02)	0.01 (−0.35, 0.37)	0.1	0.72 (0.20, 1.23) *	0.11 (−0.27, 0.50)	0.2
Log_2 Mn	−0.13 (−0.95, 0.69)	−0.85 (−1.65, −0.06) *	0.3	−0.56 (−1.35, 0.22)	0.03 (−0.82, 0.89)	0.4

Exposure	Design Fluency total correct Difference (95% CI)			Color-Word Interference completion time Difference (95% CI)		
	Males	Females	*p* for interaction	Males	Females	*p* for interaction
Log_2 DDE	0.21 (−0.26, 0.68)	−0.05 (−0.50, 0.39)	0.4	0.04 (−0.39, 0.46)	0.09 (−0.32, 0.51)	0.8
Log_2 HCB	−0.29 (−0.80, 0.22)	0.76 (0.29, 1.22) *	< 0.01*	−0.05 (−0.51, 0.41)	0.07 (−0.36, 0.50)	0.5
Log_2 ΣPCB_4	0.00 (−0.52, 0.51)	−0.34 (−0.82, 0.14)	0.5	−0.36 (−0.83, 0.10)	−0.01 (−0.45, 0.44)	0.2
Log_2 Pb	0.31 (−0.28, 0.91)	−0.05 (−0.42, 0.31)	0.3	0.08 (−0.46, 0.62)	0.01 (−0.33, 0.35)	0.9
Log_2 Mn	−0.57 (−1.47, 0.33)	0.45 (−0.36, 1.25)	0.1	−0.55 (−1.36, 0.27)	−0.46 (−1.20, 0.29)	1.0

[1]Exposures have been log2-transformed and models have been adjusted for child race, sex, age at exam, year of birth, and HOME score; maternal marital status at child's birth, IQ, seafood consumption during pregnancy, and smoking during pregnancy; maternal and paternal education and annual household income at child's birth; and study examiner. [2]Main analysis group: complete outcome, covariate and prenatal exposure biomarker data for DDE, HCB, ΣPCB_4, Pb and Mn. Total $n = 373$; Males $n = 179$; Females $n = 194$. *$p < 0.05$. Abbreviations: DDE: dichlorodiphenyldichloroethylene; HCB: hexachlorobenzene; ΣPCB_4: Sum of 4 PCB congeners (118, 138, 153, 180); Pb: lead; Mn: manganese.

Table S3. Prenatal social disadvantage index (PNSDI)[1]-stratified inverse probability weighted results of multivariable linear regression analyses (difference in scaled scores associated with a twofold increase in exposure and 95% CI)[2] assessing the relation of prenatal exposure to a five-chemical mixture with Delis-Kaplan Executive Function System (D-KEFS) cognitive flexibility scaled scores among New Bedford Cohort adolescents in the main analysis group[3].

Exposure	Trail-Making completion time Difference (95% CI)			Verbal Fluency switching accuracy Difference (95% CI)		
	PNSDI < 3	PNSDI ≥ 3	*p* for interaction	PNSDI < 3	PNSDI ≥ 3	*p* for interaction
Log_2 DDE	−0.10 (−0.42, 0.23)	−0.42 (−1.03, 0.19)	0.2	−0.09 (−0.44, 0.25)	−0.15 (−0.72, 0.42)	0.8
Log_2 HCB	0.23 (−0.15, 0.61)	−0.19 (−0.78, 0.39)	0.1	0.06 (−0.35, 0.47)	−0.40 (−0.94, 0.15)	0.2
Log_2 ΣPCB_4	−0.10 (−0.46, 0.26)	0.46 (−0.20, 1.12)	0.2	0.09 (−0.30, 0.48)	0.10 (−0.51, 0.71)	0.8
Log_2 Pb	0.03 (−0.34, 0.40)	0.07 (−0.52, 0.66)	0.9	0.22 (−0.18, 0.62)	0.35 (−0.20, 0.89)	0.5
Log_2 Mn	−0.72 (−1.40, −0.05) *	−0.29 (−1.35, 0.77)	0.6	−0.31 (−1.04, 0.41)	−0.21 (−1.19, 0.78)	0.8
**Exposure**	**Design Fluency total correct Difference (95% CI)**			**Color-Word Interference completion time Difference (95% CI)**		
	**PNSDI < 3**	**PNSDI ≥ 3**	***p* for interaction**	**PNSDI < 3**	**PNSDI ≥ 3**	***p* for interaction**
Log_2 DDE	0.09 (−0.30, 0.47)	−0.19 (−0.73, 0.35)	0.3	0.22 (−0.09, 0.53)	−0.16 (−0.75, 0.42)	0.2
Log_2 HCB	0.60 (0.16, 1.05) *	−0.47 (−0.99, 0.06)	< 0.01*	0.12 (−0.24, 0.49)	−0.09 (−0.65, 0.47)	0.3
Log_2 ΣPCB_4	−0.30 (−0.72, 0.13)	0.39 (−0.19, 0.98)	0.1	−0.35 (−0.69, 0.00)	0.23 (−0.40, 0.86)	0.2
Log_2 Pb	−0.05 (−0.49, 0.38)	0.18 (−0.35, 0.70)	0.5	−0.17 (−0.52, 0.19)	0.23 (−0.34, 0.79)	0.3
Log_2 Mn	−0.22 (−1.02, 0.57)	−0.32 (−1.26, 0.62)	0.8	−0.60 (−1.25, 0.05)	−0.46 (−1.48, 0.55)	0.8

[1]Prenatal social disadvantage index (PNSDI) was constructed as the sum of five adverse social or economic exposures at the time of the child's birth where presence of each risk factor was assigned a value of 1, absence a value of 0: mother unmarried, mother's education as a high school graduate or less, father's education as a high school graduate or less, annual household income less than $20,000, and mother's age at birth less than 20 years. [2]Exposures have been log2-transformed and models have been adjusted for child race, sex, age at exam, year of birth, and HOME score; maternal marital status at child's birth, IQ, seafood consumption during pregnancy, and smoking during pregnancy; maternal and paternal education and annual household income at child's birth; and study examiner. [3]Main analysis group: complete outcome, covariate and prenatal exposure biomarker data for DDE, HCB, ΣPCB_4, Pb and Mn. Total $n = 373$; PNSDI < 3 $n = 241$; PNSDI ≥ 3 $n = 132$. *$p < 0.05$. Abbreviations: DDE: dichlorodiphenyldichloroethylene; HCB: hexachlorobenzene; ΣPCB_4: Sum of 4 PCB congeners (118, 138, 153, 180); Pb: lead; Mn: manganese.

Table S4. Complete-case results of negative binomial regression analyses [rate ratio (RR) and 95% CI][1] assessing the relation of prenatal exposure to a five- chemical mixture with Delis-Kaplan Executive Function System (D-KEFS) cognitive flexibility error raw scores among adolescents in the main analysis group[2].

Exposure	Trail-Making total errors RR (95% CI)	Verbal Fluency total errors RR (95% CI)	Design Fluency total errors RR (95% CI)	Color-Word Interference total errors RR (95% CI)
Log_2 DDE	1.00 (0.88, 1.14)	1.23 (1.05, 1.43) *	0.99 (0.88, 1.12)	0.98 (0.89, 1.07)
Log_2 HCB	0.99 (0.86, 1.14)	0.91 (0.77, 1.06)	1.05 (0.92, 1.18)	1.03 (0.93, 1.14)
Log_2 ΣPCB_4	1.01 (0.88, 1.17)	0.95 (0.80, 1.14)	1.00 (0.88, 1.14)	1.08 (0.97, 1.19)
Log_2 Pb	0.98 (0.86, 1.11)	1.13 (0.96, 1.34)	0.99 (0.88, 1.11)	1.09 (0.99, 1.20)
Log_2 Mn	1.30 (1.01, 1.67) *	0.79 (0.58, 1.06)	0.83 (0.66, 1.03)	0.98 (0.82, 1.17)

[1]Exposures have been log2-transformed and models have been adjusted for child race, sex, age at exam, year of birth, and HOME score; maternal marital status at child's birth, IQ, seafood consumption during pregnancy, and smoking during pregnancy; maternal and paternal education and annual household income at child's birth; and study examiner. [2]Main analysis group: complete outcome, covariate and prenatal exposure biomarker data for DDE, HCB, ΣPCB_4, Pb and Mn, $n = 373$. *$p < 0.05$. Abbreviations: DDE: dichlorodiphenyldichloroethylene; HCB: hexachlorobenzene; ΣPCB_4: Sum of 4 PCB congeners (118, 138, 153, 180); Pb: lead; Mn: manganese.

Table S5. Complete-case results of logistic regression analyses [odds ratio (OR) and 95% CI][1] assessing the relation of prenatal exposure to a five-chemical mixture with odds of poor performance on Delis-Kaplan Executive Function System (D-KEFS) overall Trail-Making and Color-Word Interference performance[2] among adolescents in the main analysis group[3].

Exposure	Trail-Making Overall Performance OR (95% CI)	Color-Word Interference Overall Performance OR (95% CI)
Log_2 DDE	1.13 (0.86, 1.48)	0.96 (0.69, 1.32)
Log_2 HCB	0.97 (0.73, 1.28)	1.00 (0.73, 1.39)
Log_2 ΣPCB_4	0.93 (0.70, 1.25)	1.54 (1.09, 2.17) *
Log_2 Pb	1.11 (0.85, 1.46)	1.34 (0.98, 1.82)
Log_2 Mn	1.52 (0.91, 2.54)	1.77 (0.99, 3.17)

[1]Exposures have been log2-transformed and models have been adjusted for child race, sex, age at exam, year of birth, and HOME score; maternal marital status at child's birth, IQ, seafood consumption during pregnancy, and smoking during pregnancy; maternal and paternal education and annual household income at child's birth; and study examiner. [2]Performance takes into account both completion time and total errors raw scores. Those in the best performance group include anyone with < median level completion time and < median total errors with the remaining observations in the poor performance group.[3]Main analysis group: complete outcome, covariate and prenatal exposure biomarker data for DDE, HCB, ΣPCB_4, Pb and Mn, n = 373. *$p < 0.05$. Abbreviations: DDE: dichlorodiphenyldichloroethylene; HCB: hexachlorobenzene; ΣPCB_4: Sum of 4 PCB congeners (118, 138, 153, 180); Pb: lead; Mn: manganese.

Table S6. Inverse probability weighted results of negative binomial regression analyses [RR (rate ratio) and 95% CI][1] assessing the relation of prenatal exposure to a five-chemical mixture with Delis-Kaplan Executive Function System (D-KEFS) cognitive flexibility error raw scores among New Bedford Cohort adolescents in the main analysis group[2].

Exposure	Trail-Making total errors RR (95% CI)	Verbal Fluency total errors RR (95% CI)	Design Fluency total errors RR (95% CI)	Color-Word Interference total errors RR (95% CI)
Log_2 DDE	1.00 (0.88, 1.13)	1.24 (1.06, 1.45) *	1.00 (0.89, 1.12)	0.98 (0.89, 1.07)
Log_2 HCB	1.00 (0.86, 1.15)	0.90 (0.77, 1.05)	1.05 (0.93, 1.19)	1.03 (0.94, 1.14)
Log_2 ΣPCB_4	1.01 (0.88, 1.16)	0.94 (0.79, 1.12)	0.99 (0.87, 1.12)	1.06 (0.96, 1.17)
Log_2 Pb	1.00 (0.88, 1.14)	1.13 (0.96, 1.34)	1.00 (0.89, 1.13)	1.10 (1.00, 1.21)
Log_2 Mn	1.26 (0.99, 1.62)	0.80 (0.59, 1.07)	0.83 (0.67, 1.04)	0.96 (0.81, 1.14)

[1]Exposures have been log2-transformed and models have been adjusted for child race, sex, age at exam, year of birth, and HOME score; maternal marital status at child's birth, IQ, seafood consumption during pregnancy, and smoking during pregnancy; maternal and paternal education and annual household income at child's birth; and study examiner. [2]Main analysis group: complete outcome, covariate and prenatal exposure biomarker data for DDE, HCB, ΣPCB_4, Pb and Mn, n = 373. *$p < 0.05$. Abbreviations: DDE: dichlorodiphenyldichloroethylene; HCB: hexachlorobenzene; ΣPCB_4: Sum of 4 PCB congeners (118, 138, 153, 180); Pb: lead; Mn: manganese.

Table S7. Inverse probability weighted results of logistic regression analyses [odds ratio (OR) and 95% CI][1] assessing the relation of prenatal exposure to a five-chemical mixture with odds of poor performance on Delis-Kaplan Executive Function System (D-KEFS) overall Trail-Making and Color-Word Interference performance[2] among New Bedford Cohort adolescents in the main analysis group[3].

Exposure	Trail-Making Overall Performance OR (95% CI)	Color-Word Interference Overall Performance OR (95% CI)
Log_2 DDE	1.12 (0.86, 1.47)	0.92 (0.67, 1.27)
Log_2 HCB	0.97 (0.74, 1.29)	1.06 (0.77, 1.45)
Log_2 ΣPCB_4	0.93 (0.70, 1.24)	1.49 (1.06, 2.10) *
Log_2 Pb	1.12 (0.85, 1.47)	1.36 (1.00, 1.85)
Log_2 Mn	1.53 (0.92, 2.57)	1.66 (0.92, 2.98)

[1]Exposures have been log2-transformed and models have been adjusted for child race, sex, age at exam, year of birth, and HOME score; maternal marital status at child's birth, IQ, seafood consumption during pregnancy, and smoking during pregnancy; maternal and paternal education and annual household income at child's birth; and study examiner. [2]Performance takes into account both completion time and total errors raw scores. Those in the best performance group include anyone with < median level completion time and < median level total errors with the remaining observations in the poor performance group.[3]Main analysis group: complete outcome, covariate and prenatal exposure biomarker data for DDE,

HCB, ΣPCB4, Pb and Mn, $n = 373$. *$p < 0.05$. Abbreviations: DDE: dichlorodiphenyldichloroethylene; HCB: hexachlorobenzene; ΣPCB4: Sum of 4 PCB congeners (118, 138, 153, 180); Pb: lead; Mn: manganese.

Table S8. Characteristics of New Bedford Cohort participants who were evaluated as adolescents and included in the secondary analysis group[1], and those who were excluded from the secondary analysis group.

Descriptive Characteristic	Secondary analysis group, *n* = 235			Excluded group, *n* = 553			
**Cognitive Flexibility Measures[2]**	***n*(%)**	**Mean ± SD**	**Range**	***n*(%)**	**Mean ± SD**	**Range**	***p*-value[3]**
Trail-Making							
Completion time scaled score	235	9.8 (2.7)	1–14	293	9.1 (2.8)	1–14	< 0.01*
Total errors	235	0.8 (1.1)	0–5	292	1 (1.3)	0–13	0.03*
Overall Trail-Making performance							
Best performance	83 (35.3)			75 (13.6)			0.02*
Poor performance	152 (64.7)			217 (39.2)			
Missing	0			261 (47.2)			
Verbal Fluency scores							
Total switching accuracy scaled score	235	9.2 (2.8)	3–17	293	9.1 (2.8)	1–17	0.6
Total errors	235	0.8 (1.2)	0–7	293	0.9 (1.2)	0–7	0.4
Design Fluency							
Total correct scaled score	235	10.0 (2.8)	2–18	293	9.7 (2.7)	2–17	0.2
Total errors	235	2.5 (3.0)	0–22	293	2.7 (3.0)	0–20	0.2
Color-Word Interference							
Completion time scaled score	235	10 (2.6)	1–15	292	9.8 (2.6)	1–15	0.2
Total errors	235	2.6 (2.5)	0–19	292	2.8 (2.4)	0–11	0.2
Overall Color-Word Interference performance							0.3
Best performance	58 (24.7)			59 (10.7)			
Poor performance	177 (75.3)			233 (42.1)			
Missing	0			261 (47.2)			
**Exposure Measures[4]**							
Cord serum DDE (ng/g)	235	0.6 (1.4)	0.02–14.9	516	0.4 (0.7)	0.0–10.2	0.04*
Cord serum HCB (ng/g)	235	0.03 (0.02)	0.0–0.1	516	0.03 (0.04)	0.0–0.7	0.2
Cord serum ΣPCB4 (ng/g)	235	0.3 (0.3)	0.01–2.3	516	0.2 (0.3)	0.01–4.4	0.2
Cord blood Pb (μg/dL)	235	1.4 (0.9)	0.0–9.4	513	1.6 (1.5)	0.01–17.4	0.01*
Cord blood Mn (μg/dL)	235	4.3 (1.6)	1.7–11.2	473	4.2 (1.9)	0.2–22.1	0.8
Maternal hair MeHg (μg/g)	235	0.6 (0.6)	0.03–3.1	276	0.6 (0.7)	0.03–9.2	0.3
Maternal toenail As (μg/g)	235	0.1 (0.1)	0.02–0.8	181	0.1 (0.1)	0.02–1.0	0.5
**Covariate Measures[5]**							
Child Characteristics							
Race/Ethnicity							< 0.01*
Non-Hispanic White	186 (79.1)			345 (62.4)			
Hispanic	16 (6.8)			73 (13.2)			
Other	33 (14.0)			133 (24.1)			
Missing	0			2 (0.4)			
Sex							0.3
Male	114 (48.5)			294 (53.2)			
Female	121 (51.5)			259 (46.8)			
Age at Exam	235	15.5 (0.6)	14.4–17.7	293	15.6 (0.6)	13.9–17.9	0.5
Home Score	235	44.4 (6.0)	27–56	256	42.8 (6.5)	21–56	< 0.01*
Year of Birth							0.03*
1993–1994	76 (32.3)			183 (33.1)			
1995–1996	104 (44.3)			196 (35.4)			
1997–1998	55 (23.4)			174 (31.5)			
Maternal Characteristics							
Marital status at birth							< 0.01*
Not married	74 (31.5)			257 (46.5)			

Married	161 (68.5)			241 (43.6)			
Missing	0			55 (9.9)			
Maternal IQ	235	100.6 (9.7)	67–124	400	96.3 (10.5)	57–126	< 0.01*
Seafood during pregnancy (serv/day)	235	0.5 (0.6)	0–5.3	398	0.6 (0.7)	0–6	0.5
Smoking during pregnancy							0.3
No	171 (72.8)			311 (56.2)			
Yes	64 (27.2)			140 (25.3)			
Missing	0			102 (18.4)			
Maternal education							< 0.01*
≤ High School	108 (46.0)			313 (56.6)			
> High School	127 (54.0)			183 (33.1)			
Missing	0			57 (10.3)			
Household Characteristics at Birth							
Paternal Education							0.01*
≤ High School	152 (64.7)			360 (65.1)			
> High School	83 (35.3)			125 (22.6)			
Missing	0			68 (12.3)			
Annual Household Income							< 0.01*
< $20,000	62 (26.4)			203 (36.7)			
≥ $20,000	173 (73.6)			286 (51.7)			
Missing	0			64 (11.6)			
Examination Characteristics							
Examiner							0.3
1	171 (72.8)			227 (41.0)			
2	64 (27.2)			66 (11.9)			
Missing	0			260 (47.0)			

[1]Secondary analysis group: complete outcome, covariate and prenatal exposure biomarker data for DDE, HCB, ΣPCB_4, Pb Mn, MeHg, and As, $n = 235$. [2]NBC participants with missing cognitive flexibility measures: Trail-making completion time $n = 260$; Trail-making total errors $n = 261$; Verbal Fluency switching accuracy $n = 260$; Verbal Fluency total errors $n = 260$; Design Fluency total correct $n = 260$; Design Fluency total errors $n = 260$; Color-Word Interference completion time $n = 261$; Color-Word Interference total errors $n = 261$. [3]P-values represent results comparing characteristics between participants included in the secondary analysis group and those excluded from the secondary analysis group using t-tests, chi-square, and Wilcoxon rank sum tests, where appropriate. *p*-values reflect comparisons between groups with non-missing data. [4]NBC participants with missing exposure measures: DDE $n = 37$; HCB $n = 37$; ΣPCB_4 $n = 37$; Pb $n = 40$; Mn $n = 80$; MeHg $n = 277$; As $n = 372$. [5]NBC participants with missing covariate measures: age at exam $n = 260$; HOME score $n = 297$; maternal IQ $n = 153$; seafood during pregnancy $n = 155$. *$p < 0.05$. Abbreviations: DDE: dichlorodiphenyldichloroethylene; HCB: hexachlorobenzene; ΣPCB_4: Sum of 4 PCB congeners (118, 138, 153, 180); Pb: lead; Mn: manganese; MeHg: methylmercury; As: arsenic.

Table S9. Complete-case results of multivariable linear regression analyses (difference in scaled scores associated with a twofold increase in exposure and 95% CI)[1] assessing the relation of prenatal exposure to a seven-chemical mixture with Delis-Kaplan Executive Function System (D-KEFS) cognitive flexibility scaled scores among New Bedford Cohort adolescents in the secondary analysis group[2].

Exposure	Trail-Making completion time Difference (95% CI)	Verbal Fluency switching accuracy Difference (95% CI)	Design Fluency total correct Difference (95% CI)	Color-Word completion time Difference (95% CI)
Log_2 DDE	-0.03 (-0.44, 0.37)	0.05 (-0.37, 0.48)	-0.12 (-0.55, 0.32)	0.07 (-0.34, 0.49)
Log_2 DDE^2	-	-	-	0.16 (0.04, 0.27) *
Log_2 HCB	0.14 (-0.24, 0.53)	0.00 (-0.41, 0.41)	0.23 (-0.19, 0.65)	0.11 (-0.27, 0.50)
Log_2 ΣPCB_4	-0.06 (-0.51, 0.40)	-0.32 (-0.80, 0.16)	-0.05 (-0.54, 0.44)	-0.57 (-1.02, -0.12) *
Log_2 Pb	0.02 (-0.37 0.40)	0.26 (-0.14, 0.66)	-0.07 (-0.48, 0.34)	-0.01 (-0.39, 0.37)
Log_2 Mn	-0.53 (-1.24, 0.17)	-0.47 (-1.21, 0.28)	0.01 (-0.76, 0.78)	-0.39 (-1.09, 0.31)
Log_2 Mn^2	-	-	-1.58 (-2.63, -0.54) *	-
Log_2 MeHg	-0.09 (-0.45, 0.26)	0.22 (-0.16, 0.59)	-0.04 (-0.43, 0.34)	0.18 (-0.17, 0.53)
Log_2 As	0.10 (-0.24, 0.45)	0.17 (-0.19, 0.53)	0.05 (-0.32, 0.42)	0.02 (-0.32, 0.36)

[1]Exposures have been log2-transformed and models have been adjusted for child race, sex, age at exam, year of birth, and HOME score; maternal marital status at child's birth, IQ, seafood consumption during pregnancy, and smoking during pregnancy; maternal and paternal education and annual household income at child's birth; and study examiner. [2]Secondary analysis group: complete outcome, covariate and prenatal exposure biomarker data for DDE, HCB, ΣPCB_4, Pb, Mn, MeHg, and As, n = 235. *$p < 0.05$. Abbreviations: DDE: dichlorodiphenyldichloroethylene; HCB: hexachlorobenzene; ΣPCB_4: Sum of 4 PCB congeners (118, 138, 153, 180); Pb: lead; Mn: manganese; MeHg: methylmercury; As: arsenic.

Table S10. Inverse-probability weighted results of multivariable linear regression analyses (difference in scaled scores associated with a twofold increase in exposure and 95% CI)[1] assessing the relation of prenatal exposure to a seven-chemical mixture with Delis-Kaplan Executive Function System (D-KEFS) cognitive flexibility scaled scores among New Bedford Cohort adolescents in the secondary analysis group[2].

Exposure	Trail-Making completion time Difference (95% CI)	Verbal Fluency switching accuracy Difference (95% CI)	Design Fluency total correct Difference (95% CI)	Color-Word completion time Difference (95% CI)
Log_2 DDE	-0.12 (-0.53, 0.30)	0.06 (-0.36, 0.48)	-0.18 (-0.61, 0.24)	-0.06 (-0.50, 0.38)
Log_2 DDE^2	-	-	-	0.17 (0.06, 0.29) *
Log_2 HCB	0.10 (-0.29, 0.50)	-0.11 (-0.51, 0.29)	0.18 (-0.23, 0.58)	0.10 (-0.30, 0.50)
Log_2 ΣPCB_4	0.02 (-0.46, 0.50)	-0.40 (-0.89, 0.08)	0.01 (-0.48, 0.50)	-0.51 (-1.00, -0.03) *
Log_2 Pb	-0.02 (-0.42, 0.39)	0.35 (-0.06, 0.77)	-0.01 (-0.43, 0.40)	0.00 (-0.41, 0.41)
Log_2 Mn	-0.39 (-1.14, 0.35)	-0.33 (-1.08, 0.42)	-0.08 (-0.87, 0.70)	-0.33 (-1.08, 0.43)
Log_2 Mn^2	-	-	-1.50 (-2.55, -0.45) *	-
Log_2 MeHg	-0.04 (-0.42, 0.35)	0.27 (-0.12, 0.65)	-0.12 (-0.51, 0.27)	0.20 (-0.19, 0.59)
Log_2 As	0.08 (-0.29, 0.46)	0.17 (-0.21, 0.55)	0.13 (-0.25, 0.52)	-0.03 (-0.41, 0.35)

[1]Exposures have been log2-transformed and models have been adjusted for child race, sex, age at exam, year of birth, and HOME score; maternal marital status at child's birth, IQ, seafood consumption during pregnancy, and smoking during pregnancy; maternal and paternal education and annual household income at child's birth; and study examiner. [2]Secondary analysis group: complete outcome, covariate and prenatal exposure biomarker data for DDE, HCB, ΣPCB_4, Pb, Mn, MeHg, and As, n = 235. *$p < 0.05$. Abbreviations: DDE: dichlorodiphenyldichloroethylene; HCB: hexachlorobenzene; ΣPCB_4: Sum of 4 PCB congeners (118, 138, 153, 180); Pb: lead; Mn: manganese; MeHg: methylmercury; As: arsenic.
